# Hypoxia-hindered methylation of PTGIS in endometrial stromal cells accelerates endometriosis progression by inducing CD16^−^ NK-cell differentiation

**DOI:** 10.1038/s12276-022-00793-1

**Published:** 2022-07-04

**Authors:** Haiyan Peng, Lichun Weng, Shating Lei, Shuhui Hou, Shaoliang Yang, Mingqing Li, Dong Zhao

**Affiliations:** 1 Shanghai Key Laboratory of Maternal Fetal Medicine, Clinical and Translational Research Center, Shanghai First Maternity and Infant Hospital, School of Medicine, Tongji University, Shanghai, 200092 China; 2 Laboratory for Reproductive Immunology, Shanghai Key Laboratory of Female Reproductive Endocrine Related Diseases, Obstetrics and Gynecology Hospital of Fudan University, Shanghai, 200011 China; 3 Key Laboratory of Reproduction Regulation of NPFPC, SIPPR, IRD, Fudan University, Shanghai, 200032 China; 4 Present Address: Department of Obstetrics and Gynecology, Shanghai Ninth People’s Hospital, School of Medicine, Shanghai Jiao Tong University, Shanghai, 200011 China

**Keywords:** Immunological disorders, DNA methylation

## Abstract

Prostacyclin (PGI_2_) plays key roles in shaping the immune microenvironment and modulating vasodilation, whereas its contribution to endometriosis (EMs) remains largely unclear. Our study suggested that prostacyclin synthase (PTGIS)-dependent PGI_2_ signaling was significantly activated in EMs, which was involved in the hypoxic microenvironment of ectopic lesions and deficient methylation status of the *PTGIS* promoter. Notably, in vitro assays, hypoxia promoted PTGIS expression through DNA methyltransferase 1 (DNMT1)-mediated DNA methylation deficiency in endometrial stromal cells (ESCs); PTGIS overexpression enhanced the adhesive ability of ESCs and led to elevated PGI_2_ production, and PGI_2_ triggered CD16^−^ (encoded by *FCGR3*, Fc fragment of IgG receptor IIIa) natural killer (NK)-cell differentiation through PGI_2_ receptor (IP, PTGIR) in an ESC/NK-cell coculture system. Our rodent model experiment suggested that treatment with the PGI_2_ analog iloprost and adoptive transfer of *fcgr3* knockout (*fcgr3*^−/−^) NK cells aggravated EMs progression and that genetic ablation of *ptgis* (*ptgis*^−/−^) in ectopic lesions and treatment with the PTGIR antagonist RO1138452 partially rescued this outcome. Thus, our findings identified the contribution of PGI_2_ to EMs progression via enhancement of the adhesive ability of ESCs and inhibition of the activity of NK cells. We hypothesized that PGI_2_ is a target for EMs intervention and provide a rationale for studying pharmacological PTGIR inhibition and *PTGIS* genetic depletion therapies as therapeutic strategies for EMs.

## Endometriosis: Enzyme could hold the key to novel therapeutics

Inhibiting the activity of a critical enzyme found overexpressed in endometriosis lesions could lead to novel therapeutics. Endometriosis affects around 10 per cent of women of reproductive age globally, yet the condition is poorly understood. Endometriosis lesions are known to be in a hypoxic, or low oxygen, state. Zhao Dong at Tongji University in Shanghai, China, and co-workers used human tissue samples and mouse models to determine the roles of a metabolite called prostacyclin (PGI_2_) and its catalytic enzyme (prostacyclin synthase, PTGIS) in endometriosis. PTGIS levels were significantly elevated in hypoxic endometrial cells, triggering the overproduction of PGI_2_. This PTGIS/PGI_2_ increase enhanced the adhesiveness of the cells, promoting survival of developing lesions. PGI_2_ overproduction also triggered abnormal differentiation of a specific group of immune cells called natural killer cells, disrupting the body’s immune response.

## Introduction

Endometriosis (EMs) is an enigmatic benign estrogen-dependent gynecological disease characterized by viable endometrial glands and stroma survived outside the uterine cavity; EMs is predominantly, but not exclusively, present in the ovaries and affects approximately 10% of women of reproductive age worldwide^1–3^. Accumulating evidence suggests that EMs is a multigenic disorder^4,5^, and intrinsic disorder in endometrial stromal cells (ESCs) and dysfunctional immunosuppression serve as two crucial contributors to its prevalence^6–8^; however, the underlying pathogenesis remains to be elucidated. Prostaglandins are the metabolic products of arachidonic acid metabolism, namely, PGD_2_, PGE_2_ and PGF_2α_, and PGE_2_ has been shown to promote inflammatory cell infiltration, cellular phenotype modulation and immunity dysregulation in EMs patients^9–11^. In contrast, other analogs of PGE_2_ have rarely been investigated in EMs, such as prostacyclin (PGI_2_). PGI_2_ is predominantly expressed in the vascular endothelium, and its biosynthesis is catalyzed by the rate-limiting enzyme prostacyclin synthase (PTGIS, PGIS or CYP8A1), which is a member of family 8 (CYP8) in the cytochrome P450 superfamily. Based on previous publications, the biological function of PGI_2_ was quite different from that of PGE_2_, and PGI_2_ was proposed to be a vasodilator and has been applied to treat pulmonary arterial hypertension (PAH) in the clinic^12–16^. In addition, PGI_2_ profoundly modulates immune responses mediated by regulatory T cells, natural killer (NK) cells, immature dendritic cells, B cells and CD4^+^ T-cell subsets in diseases^17–25^, and the concentration of PGI_2_ was reported to be increased in the peritoneal fluid of EMs patients^26^, whereas the role of PGI_2_ in EMs remains unknown.

CD3^−^CD56^+^ NK cells play pivotal roles in innate immunity, and NK cells with a CD16^+^ (encoded by *FCGR3*, Fc fragment of IgG receptor IIIa) phenotype are naturally cytotoxic and mainly exert killing effects, while CD16^−^ NK cells were efficient cytokine producers endowed with immunoregulatory properties that can become cytotoxic upon activation^27^. In general, CD16 expression is downregulated following NK-cell activation; CD16 is not only an activating receptor of NK cells but also matters in disease progression. Remarkably, CD16^−^ NK-cell infiltration has been reported to be profoundly associated with papillary thyroid cancer progression^28^, with these cells demonstrating an impaired capability to kill non-small cell lung cancer cells^27^ and displaying an enhanced antioxidative capacity^29^. Additionally, CD56^dim^CD16^−^ NK-cell subsets are increased in the peripheral blood of advanced invasive breast cancer patients^30^. We reported an increased proportion of CD16^−^ NK cells in the peritoneal fluid of EMs patients, which served as an active mediator for promoting EMs development; however, the underlying mechanism of this abnormal differentiation is not fully defined^31^. Bryan Simons reported that PGI_2_ was also endowed with the ability to modulate the number and properties of NK cells resident in the respiratory tract, relating to allergic asthma^19^, suggesting that PGI_2_ might correlate with the abnormal NK-cell differentiation in EMs. Therefore, our study focused on analyzing whether the augmented CD16^−^ NK-cell population in EMs resulted from PTGIS/PGI_2_ signaling in ESCs, aiming to provide a theoretical foundation for developing new approaches to combat this disease.

## Materials and methods

### Cells and culture

A human endometrial stromal cell (HESC) line was purchased from the Cell Bank of the Chinese Academy of Sciences (Shanghai, China), and NK92 cells were a generous gift from Professor Qiang Fu at Binzhou Medical University. HESC and primary ESCs were cultured in DMEM/F12 (HyClone, USA) supplemented with 10% fetal bovine serum (FBS) (ScienCell, USA) and a 1% penicillin–streptomycin–amphotericin B solution (NCM, Suzhou). Human peripheral blood NK cells were cultured in RPMI 1640 medium (HyClone) supplemented with FBS and antibiotics. NK92 cells were cultured in serum-free medium for lymphocytes (DAKEWE, Shenzhen) supplemented with 20% FBS and 1% antibiotics. All cells were cultured in an incubator with 5% CO_2_ at 37 °C.

### Clinical specimen collection and isolation of primary ESCs

This study was approved by the Ethics Committee of Tongji University School of Medicine affiliated Shanghai First Maternity and Infant Hospital. In total, 53 ovarian EMs patients and 47 tubal infertility, leiomyoma, or benign ovarian cyst patients provided informed consent were enrolled in the study, and none of the patients had used hormonal contraceptives in the past 6 months. Twenty-four cases of ovarian endometriotic cyst tissues (defined as ectopic endometrium), 20 cases of normal endometrium specimens and 56 cases of peritoneal fluid samples were collected during surgical procedures, and the menstrual cycle phase of each specimen was further verified by experienced histopathologists. The disease stage of EMs patients was defined according to the American Society of Reproductive Medicine (ASRM) revised classification system, and the clinical characteristics of the patients are summarized in Table 1 and Supplementary Table 1. The endometriotic lesions of EMs patients and normal endometrial tissues are collected into culture medium and immediately transferred to the laboratory on ice. Each collected biopsy was divided into 3 parts: one was frozen in liquid nitrogen, one was stored in 4% formalin (Biotech Well, Shanghai) for immunohistochemical analysis, and one was dissected, cut into pieces and digested with collagenase IV (Sigma, USA) at 37 °C for 30 min (min). The digested samples were filtered through a 70-μm strainer (FALCON, USA) and centrifuged at 1000 rpm for 10 min. Then, the cell pellets were resuspended and cultured in medium in an incubator. Primary ESCs were confirmed by detecting the expression levels of vimentin and cytokeratin 7. In addition, 29 endometriotic and 27 control peritoneal fluid samples were centrifuged at 3000 rpm for 20 min, and the supernatants were stored at −80 °C until further analyzed.

**Table 1:** Patient demographic profile.

Group	EMs^a^	Control	Endometrium		Isolated ESCs^b^		Peritoneal fluid	
Numbers	53	47	24 (E^c^)	20 (N^d^)	12 (E)	12 (N)	29 (E)	27 (N)
Age (Y)^e^	34.55 ± 0.89	35.47 ± 1.04	34.25 ± 1.40	37.40 ± 1.59	35.75 ± 2.27	36.83 ± 2.42	34.79 ± 1.15	34.04 ± 1.33
*P* Value	0.4996		0.1435		0.7472		0.6686	
Cycle Phase								
Proliferative	41	35	20	15	9	10	21	20
Secretory	12	12	4	5	3	2	8	7
Dysmenorrhea								
No	29	32	14	16	8	10	15	16
Mild	12	5	5	1	3	1	7	4
Moderate	10	8	5	3	1	1	5	5
Severe	2	2	0	0	0	0	2	2
Stage (rASRM^f^)								
Stage I–II	13		5		4		8	
Stage III–IV	40		19		8		21	

^a^EMs: endometriosis.

^b^ESCs: endometrial stromal cells.

^c^E: endometriosis patients.

^d^N: Control patients.

^e^Age (Y): mean ± standard error of the mean (SEM) (years).

^f^rASRM: Revised American Society for Reproductive Medicine classification.

### Animals and EMs models

All in vivo experiments in the study were approved by the Animal Care Committee of Tongji University. Wild-type (WT) female mice on the C57BL/6 J (C57) background (aged 6–8 weeks (w)) were purchased from Shanghai Jiesjie Laboratory Animal Co., Ltd. *Ptgis*^−^^/−^ mice and *fcgr3*^−^^/−^ mice on the C57 background were constructed, bred and fed in an SPF environment by Shanghai Model Organisms Center, Inc. The methods for establishment the syngeneic mouse EMs model were previously described and slightly improved^31^. Briefly, ~6 to 8 w-old female donor and recipient mice were intramuscularly injected with 3 μg/mouse 17 β-estradiol (Sigma) on Day 1 and Day 4. Then, the donor mice were euthanized, and the uterus was collected and dissected into 1-mm^3^ fragments on Day 7. The uterine fragments with endometrial tissue in the proliferative phase were resuspended in PBS and injected into the peritoneal cavity of recipient mice, and when the endometrial debris was implanted, the EMs models were successfully established. In some groups of EMs model mice, drugs or PBS were injected intraperitoneally daily for 10 days beginning on Day 10. On Day 21, the EMs model mice were sacrificed, and we observed, collected and reserved the endometriosis-like lesions implanted in the peritoneal cavity.

### GEO database bioinformatic analysis

EMs-related Gene Expression Omnibus (GEO) datasets (GSE23339, GSE7305, GSE5108, GSE25628, GSE11691, GSE78851, GSE12768, GSE6364, GSE94414, GSE58178, GSE87809, GSE31515, GSE47360, GSE73950, GSE47361, and GSE87621), consisting of several types of EMs, were downloaded from the website (https://www.ncbi.nlm.nih.gov/gds/?term=endometriosis); these datasets were uploaded and generously shared by an increasing number of researchers in related fields. The samples in these datasets were mainly ectopic endometriotic lesion biopsies, normal endometrial tissues and isolated ESCs, and more detailed information is summarized in Table 2. We determined the original expression and methylation status of *PTGIS* in all samples according to the annotations of relative platforms when the datasets were complied with a standard. The data we used were mainly generated with two approaches: RNA-sequencing or gene microarray and DNA methylation sequencing. All data were standardized and further analyzed by statistical analysis.

**Table 2:** Characteristics of GEO datasets.

Dataset	Platform	Positions	Category	Type	Sample size
GSE23339	GPL6102	Ovarian	Transcription	Tissue	N (9) vs. OE (10)
GSE7305	GPL570	Ovarian	Transcription	Tissue	N (10) vs. OE (10)
GSE5108	GPL2895	Ovarian & Peritoneal	Transcription	Tissue	N (10) vs. OE (8) vs. PE (3)
GSE25628	GPL571	Deep Infiltrating EMs	Transcription	Tissue	N (6) vs. DEU (8) vs. DEC (8)
GSE11691	GPL96	Broad Ligament & Peritoneal	Transcription	Tissue	N (9) vs. BE (7) vs. PE (2)
GSE78851	GPL6244	Adenomyosis	Transcription	Tissue	N (5) vs. AE (3)
GSE12768	GPL7304	Ovarian	Transcription	Tissue	N (2) vs. OE (2)
GSE6364	GPL570	Ovarian	Transcription	Tissue	N (16) vs. OE (21)
GSE94414	GPL22166	Ovarian, Peritoneal & Retrocervical	Transcription	Tissue	N (6) vs. OE (4) vs. PE (4) vs. RE (1)
GSE58178	GPL6947	Ovarian	Transcription	ESCs	N (6) vs. OE (6)
GSE87809	GPL11154	Ovarian	Transcription	ESCs	N (5) vs. OE (4)
GSE31515	GPL6480	Ovarian	Transcription	ESCs	N (3) vs. OEU (3) vs. OEC (3)
GSE47360	GPL6244	Ovarian	Transcription	ESCs	N (3) vs. OEU (3) vs. OEC (3)
GSE73950	GPL13534	Ovarian	DNA Methylation	Tissue	N (17) vs. OEU (26)
GSE47361	GPL6244	Ovarian	DNA Methylation	ESCs	N (3) vs. OEU (3) vs. OEC (3)
GSE87621	GPL13534	Ovarian	DNA Methylation	ESCs	N (5) vs. OE (4)

*N* normal, *OE* ovarian endometriosis, *PE* peritoneal endometriosis, *DEU* eutopic endometrium of deep infiltrating endometriosis, *DEC* ectopic endometrium of deep infiltrating endometriosis, *BE* broad ligament endometriosis, *AE* adenomyosis, *RE* retrocervical, *OEU* eutopic endometrium of ovarian endometriosis, *OEC* ectopic endometrium of ovarian endometriosis.

### Lentiviral transfection

The lentiviruses CON077, LV-HIF-1A-RNAi, LV-DNMT1-RNAi (hU6-MCS-Ubiquitin-EGFP-IRES-puromycin), CON238, LV-PTGIS and LV-DNMT1 (Ubi-MCS-3FLAG-SV40-EGFP-IRES-puromycin) were all constructed by GENECHEM (Shanghai). HESCs were treated with trypsin containing 0.25% EDTA (NCM), centrifuged and cultured in 6-well plates (Corning, USA). Then, a lentivirus and transfection enhancer reagent were added to the culture medium when the cells reached 50% confluence, and the medium was exchanged 12 h (h) after transfection. After transfection for 72 h, the HESCs were inspected by fluorescence microscopy, and the transfection efficiency was verified by qRT–PCR and western blotting assays. Then, the cells were treated with 2 μg/ml puromycin (MCE, China) for 7 days to select the successfully transfected cells.

### Human peripheral blood and mouse spleen NK-cell isolation

Normal peripheral blood was extracted from a vein in the elbow of female volunteers. Then, peripheral blood mononuclear cells (PBMCs) were separated with Ficoll (GE Healthcare, USA); we reserved the layer containing these cells and washed it with sterile PBS 3 times. Peripheral NK cells (pNK cells) were isolated from the PBMCs with a human NK-cell isolation kit via negative selection (STEMCELL, Canada) and further cultured in RPMI 1640 (HyClone) medium supplemented with FBS and antibiotics. To isolate NK cells from the mouse spleen, ~6- to 8-w-old female mice on the C57 background were sacrificed, and their spleens were taken out. We cut the spleens into pieces, removed the debris with 40-μm filters (FALCON) and centrifuged the cell suspensions at 1000 rpm for 10 min. Then, red blood cell lysis buffer (BioLegend, China) was used to lyse the red blood cells. Then, all the cells were incubated with reagents from a mouse NK-cell isolation kit (Miltenyi, Germany) and passed through a magnetic column (Miltenyi), and the mouse NK cells were isolated. The purity of isolated human pNK cells and mouse spleen NK cells was evaluated by flow cytometric analysis, and cells were used in further studies when their purity was greater than 90%.

### RNA extraction and quantitative Real-Time PCR (qRT–PCR)

Tissue and cell samples were treated with TRIzol (TAKARA, Japan) for 15 min on ice, and RNA was extracted with traditional extraction methods. After the RNA samples were quantified and qualified, they were used as templates for the reverse transcription of complementary DNA (cDNA), with a total of 1000 ng RNA used for each sample. The reaction system was processed at 37 °C for 15 min, 85 °C for 5 s (s) and 4 °C indefinitely. Then, the products were evaluated by real-time PCR; the reaction system was set and processed according to the manufacturer’s suggestions as follows: 95 °C for 30 sec for stage 1; 95 °C for 5 s and 60 °C for 30 s, repeated for 40 cycles for stage 2; and 95 °C for 15 s, 60 °C for 1 min, and 95 °C for 15 s for the melting curve stage. The primers used are shown in Table 3 and were constructed by Sangon Biotech (Shanghai), and the data were further analyzed to calculate relative gene expression values.

**Table 3:** Sequences of primers used for qRT–PCR.

Gene Name	Forward or Reverse	Sequence (5′-3′)
HIF-1α	Forward	GAACGTCGAAAAGAAAAGTCTC
	Reverse	CCTTATCAAGATGCGAACTCACA
PTGIS	Forward	ATGCCTGCGAGAGACCCTACA
	Reverse	GCAAGTCACCTCACCTCTCAGTT
DNMT1	Forward	AACCTTCACCTAGCCCCAG
	Reverse	CTCATCCGATTTGGCTCTTTCA
DNMT3A	Forward	CAGCTTCCACGTTGCCTTCT
	Reverse	CATCTGCAAGCTGTCTCCCTTT
DNMT3B	Forward	AGGGAAGACTCGATCCTCGTC
	Reverse	GTGTGTAGCTTAGCAGACTGG
β-actin	Forward	TGGCACCCAGCACAATGAA
	Reverse	CTAAGTCATAGTCCGCCTAGAAGCA
GAPDH	Forward	GCCTCAAGATCATCAGCAATGCCT
	Reverse	GTGGTCATGAGTCCTTCCACGAT

### Immunohistochemical (IHC) staining and semiquantitative analysis

Collected normal and ectopic endometrial tissues were fixed with 4% formalin (Biotech Well) for 12 h at 4 °C, embedded in paraffin and cut into 5-µm-thick slices. Then, the specimens underwent dewaxing, dehydration and antigen retrieval, and they were further incubated with primary antibodies against PTGIS and HIF-1α (Abcam, UK) in a humidified box overnight at 4 °C. The following day, the specimens were washed with PBS 3 times and then incubated with an HRP-conjugated goat anti-rabbit antibody (Abcam) for 30 min at room temperature. Then, all slides were reacted with DAB substrate and hematoxylin dye reagents (Biotech Well). Rabbit-derived isotype-matched immunoglobulin G was incubated with slides used as negative controls. Detailed information on the antibodies and reagents is listed in Supplementary Table 2. Finally, the specimens were observed and imaged randomly under a microscope. The staining intensity of the slides was examined and verified independently by two observers, and scores were calculated as the percentage of positive immunoreactive cells. No staining was recorded as no staining; staining of less than 10% was defined as weak staining; staining of 11–50% was defined as moderate staining; and staining of more than 50% was defined as strong staining.

### Western blotting

Tissues and cells were washed with precooled PBS and treated with RIPA buffer (Beyotime, Shanghai) on ice for 30 min. Total protein was collected from the cell lysates and centrifuged at 4 °C and 12000 rpm for 20 min, and the supernatants of all lysates were reserved. Then, we determined the protein concentration of all samples by BCA methods (Beyotime). Loading buffer was added to the protein samples, and they were heated at 4 °C for 10 min. Then, all samples were ready for use in further studies. After a SDS–PAGE gel (Beyotime) was prepared, 25 μg of protein sample was added per well for each sample. By electrophoresis, the proteins with different molecular weights were separated in the gel, and then they were transferred to a PVDF membrane (Millipore, USA). The membrane was incubated with a primary antibody at 4 °C overnight. On the following day, after being washed with TBST (Beyotime) 3 times, the PVDF membrane was incubated with an HRP-conjugated secondary antibody for 1 h at room temperature on an orbital shaker. Finally, the PVDF membrane was exposed to light for imaging with immobilon western chemiluminescence HRP substrate (Millipore), and the relative intensity of the protein band was determined with Image-Pro Plus software version 6.0 (Media Cybernetics, Rockville, MD, USA).

### Flow cytometric analysis (FCM)

Cells were trypsinized with 0.25% non-EDTA trypsin and centrifuged at 1000 rpm for 10 min. Then, the cells were resuspended in precooled PBS, and a single-cell suspension was prepared for detecting surface and intracellular molecules. To determine the expression level of cell-surface molecules, cells were directly incubated with a fluorophore-conjugated antibody (BioLegend) for 30 min at room temperature protected from light, with negative control cells incubated with isotype reagents. To stain for intracellular molecules, cells underwent fixation and permeabilization processes and were then incubated with a fluorophore-conjugated antibody. Thirty minutes later, the cells were washed with PBS and centrifuged at 1000 rpm for 5 min. Then, the cell pellets were resuspended in 300 μl PBS and further detected on a flow cytometer machine, and the expression level of each molecule was calculated according to the mean fluorescence intensity.

### Immunofluorescence assay

For evaluation by immunofluorescence staining, cells were digested, plated in dishes (3.5 cm in diameter) (NEST, USA) and cultured in a 5% CO_2_, 37 °C atmosphere for 24 h. Then, as the cells were attached to the bottom of the dishes, we removed the culture medium, washed the cells with PBS 3 times and fixed them with 4% paraformaldehyde (Biotech Well) for 10 min. After permeabilization and blocking processes, the cells were incubated with a diluted primary rabbit antibody (Abcam) at 4 °C overnight, with negative control cells incubated with PBS. On the following day, the antibody was recycled, and the cells were washed with PBS 3 times and then incubated with a fluorescent HRP-conjugated goat anti-rabbit antibody (Abcam) at room temperature for 1 h protected from light. One hour later, we removed the secondary antibody, washed the cells with PBS 3 times and then stained them with DAPI reagent (Biotech Well) for 5 min in the dark. Finally, the cells were inspected by confocal microscopy and imaged.

### Enzyme-linked immunosorbent assay (ELISA)

The level of 6-keto prostaglandin F_1α_ was determined by ELISA (Cayman, USA); 6-keto prostaglandin F_1α_ is a stable metabolite of PGI_2_ and can be detected as an indicator of the PGI_2_ level. The supernatants of cells, tissue lysates and peritoneal fluid from volunteers were collected and centrifuged at 3000 rpm for 20 min. Then, we collected the supernatants for further detection. An ELISA kit was removed from the refrigerator and allowed to warm to room temperature. When all reagents were prepared, we added standard reagents, supernatant samples or PBS (used as a negative control) to the wells of a 96-well plate and incubated the plate for 18 h at 4 °C. The next day, the 96-well plate was removed from the refrigerator, and we removed the supernatants, washed the plate thoroughly with wash buffer 5 times and added reconstituted Ellman’s reagents. Then, the plate was developed on an orbital shaker protected from light for 90 min at room temperature according to the manufacturer’s instructions. Finally, the optical density in the wells of the plate was detected at a wavelength of 420 nm on a microplate reader, and we determined the PGI_2_ concentrations of all samples according to the standard curve.

### DNA methylation sequencing

To determine the methylation status of a PTGIS gene reporter, we performed functional promoter sequence analysis. Then, we verified the DNA methylation status of the PTGIS gene promoter in two groups of isolated primary ESCs by performing genomic bisulfite sequencing and dissociation curve analysis. The procedure was performed as follows: we extracted DNA from 8 cases of nESCs and 8 cases of eESCs, and the DNA was treated with bisulfite. Then, the products further underwent PCR process, and the PCR primers are listed in Table 4. Samples were detected in triplicate, and we sequenced 8–10 individual clones from each reaction. Then, the methylation data were collected and analyzed.

**Table 4:** Sequences of primers used to detect the methylation status of the PTGIS promoter.

Purpose	Sequence (5′-3′)
PTGIS CpG island, bisulfite sequencing, first PCR	TTTTAAARTGGGTTGGGGTGGGCCTTCCCACCTTACACCTTCTTA
PTGIS CpG island, bisulfite sequencing, nested PCR	GGAATTTTATTTGGGAGTGGGTTCACCTTCTTAACAAAAAAAAC

### Adhesion assay

Negative control CON238 HESCs and LV-PTGIS HESCs were digested with 0.25% EDTA-trypsin, and then the cells were resuspended and centrifuged at 1000 rpm for 10 min. The cell pellets were resuspended in culture medium. These two groups of HESCs were counted and plated in 24-well plates in triplicate (5 × 10^5^ cells per well). Twenty-four hours later, when the cells had attached to the bottom of the plate, the HESCs were digested, centrifuged, resuspended in medium and stained with PKH26 (Sigma). Then, we plated the cells in the previous plate to coculture them with CON238 and LV-PTGIS HESCs. The plate was then incubated in a 37 °C incubator protected from light. Thirty minutes later, we removed the culture medium, resuspended the HESCs, and gently washed the cells with sterile PBS 3 times. Then, the plate was inspected under a fluorescence microscope. We randomly acquired 5 images for each well and calculated the adhesion index of CON238 and LV-PTGIS HESCs.

### Mouse intraperitoneal NK-cell depletion and adoptive transfer of NK cells

We used an anti-NK1.1 antibody (BioLegend) to eliminate peritoneal NK cells in mouse EMs models. On Day 7 and Day 9, 200 μg anti-NK1.1 was intraperitoneally injected into EMs model mice to deplete NK cells, and negative control reagents were intraperitoneally injected into other EMs model mice. On Day 11, some of the anti-NK1.1- and negative control reagent-injected EMs model mice were sacrificed, and we injected 1 ml of PBS into the peritoneal cavity and collected the peritoneal lavage fluid. The cells in the peritoneal lavage fluid were analyzed by FCM to verify the NK-cell depletion efficiency. On the same day, donor WT and *fcgr3*^−/−^ mice were sacrificed, and we collected the spleens from the peritoneal cavity, cut them into pieces with sterile scissors, and filtered them through 40-μm filters (FALCON). Subsequently, we lysed the red blood cells, and then mouse spleen NK cells were isolated with magnetic beads using a negative selection method (Miltenyi). Isolated NK cells (5 × 10^5^) were adoptively transferred into the peritoneal cavity of each recipient EMs model mouse via intraperitoneal injection. Ten days after NK-cell adoptive transfer, the EMs model mice were all sacrificed to collect the peritoneal lavage fluid and endometriotic lesions.

### Statistical analysis

Data are presented as the mean ± standard error of the mean (SEM), and statistical analysis was performed using Student’s t test, one-way analysis of variance (one-way ANOVA) and the Mann–Whitney test with Prism version 6.0 GraphPad software (USA). A *P* value less than 0.05 was defined as significant.

## Results

### PTGIS and PGI_2_ are increased in ESCs from patients with EMs

**Fig. 1:**
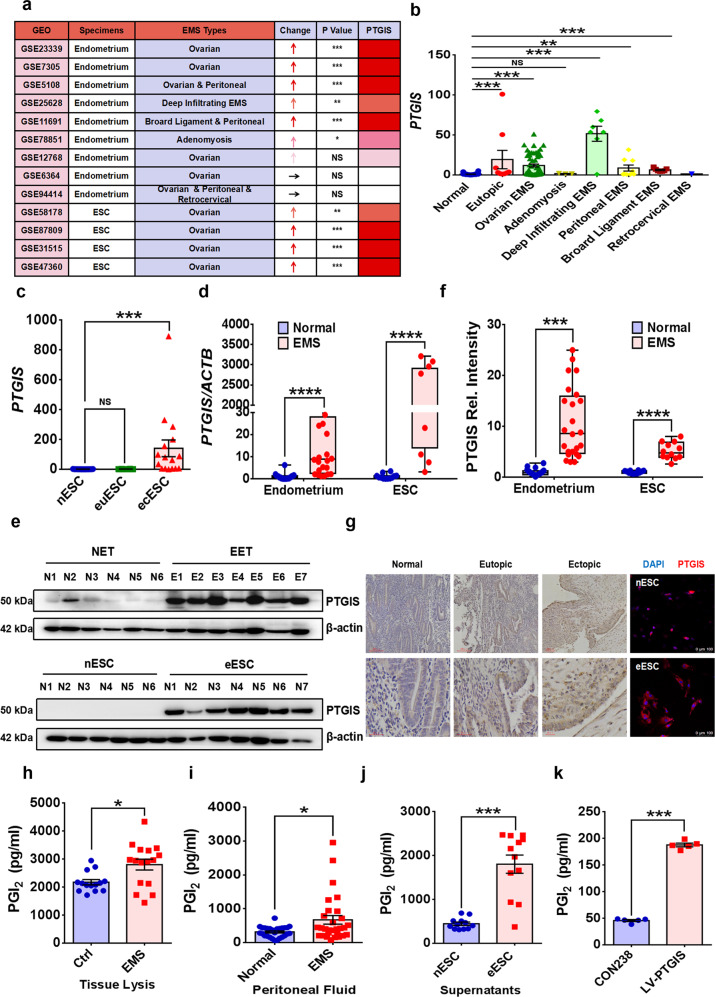
EMs specimens display considerably augmented levels of PTGIS/PGI_2_. **a** Information from GEO datasets. The expression of PTGIS was elevated in most endometriotic lesions. **b** Nearly all types of EMs specimens displayed increased levels of PTGIS. **c** Ectopic ESCs had higher levels of PTGIS than normal ESCs in GEO datasets. **d** The gene expression level of *PTGIS* was increased in endometriotic lesions (*n* = 20) compared to the normal endometrium (*n* = 24), and ectopic ESCs (*n* = 12) had higher gene levels of PTGIS than normal ESCs (*n* = 12). **e**, **f** The protein level of PTGIS was substantially increased in endometriotic lesions and eESCs. **g** IHC staining was performed with samples of normal endometrium, eutopic endometrium of EMs and ectopic lesions of EMs, and it showed that PTGIS expression was elevated in the eutopic and ectopic endometrium. An immunofluorescence assay visualized the augmented PTGIS expression in eESCs. **h**–**j** Tissue lysates of endometriotic lesions (*n* = 16), peritoneal fluid collected from EMs (*n* = 29) volunteers and supernatants of eESCs (*n* = 12) exhibited higher concentrations of PGI_2_. **k** Upregulation of PTGIS expression in HESCs encouraged overproduction of PGI_2_. Statistical analysis was performed with Student’s t test, one-way analysis of variance (ANOVA) or the Mann–Whitney test. *P < 0.05, **P < 0.01, ***P < 0.001, ****P < 0.0001. NS no significant difference, GEO Gene Expression Omnibus, EMs endometriosis, nESC normal endometrial stromal cells, euESC eutopic endometrial stromal cells, ecESC ectopic endometrial stromal cells, NET normal endometrial tissues, EET ectopic endometrial tissues, eESCs ectopic endometrial stromal cells, IHC immunohistochemistry, DAPI nuclear stain. Scale bars: 100 μm and 30 μm for IHC and 100 μm for immunofluorescence.

PGI_2_ production is specifically catalyzed by PTGIS; thus, we first investigated the expression of PTGIS in EMs samples in EMs transcriptional profile-related GEO datasets (GSE23339, GSE7305, GSE5108, GSE25628, GSE11691, GSE78851, GSE12768, GSE6364, GSE94414, GSE58178, GSE87809, GSE31515 and GSE47360), and the results revealed substantially elevated *PTGIS* levels in endometriotic lesions and isolated ovarian endometrioma ESCs (Fig. 1a). To exclude any possible bias resulting from distinctions among tissue types, samples were broadly grouped into different subgroups according to EMs localization, such as ovarian endometriotic cysts, deep infiltrating EMs (DIE) and adenomyosis. Concordant with our finding, most types of EMs exhibited observably increased levels of *PTGIS* (Fig. 1b, c). To verify the transcriptional profile of *PTGIS* identified in the GEO datasets, we collected normal endometrial tissues (nEM), endometriotic lesions (eEM) and primary ESCs from volunteers. Assays were performed to determine the expression level of PTGIS, and we observed robustly elevated levels of PTGIS in endometriotic lesions and ovarian ESCs (Fig. 1d–g), consistent with the results for the GEO datasets. Notably, EMs specimens derived from eEM lysates, peritoneal fluid samples and primary ESCs supernatants all exhibited increased concentrations of PGI_2_ (Fig. 1h–j). To examine the catalytic efficiency of PTGIS in PGI_2_ production, we upregulated the expression of PTGIS in HESCs, and the results showed that elevated PTGIS enabled overproduction of PGI_2_ (Fig. 1k, Supplementary Fig. 1a, b).

### Hypoxia upregulates PTGIS/PGI_2_ expression in ESCs by altering the methylation status of the PTGIS promoter

**Fig. 2:**
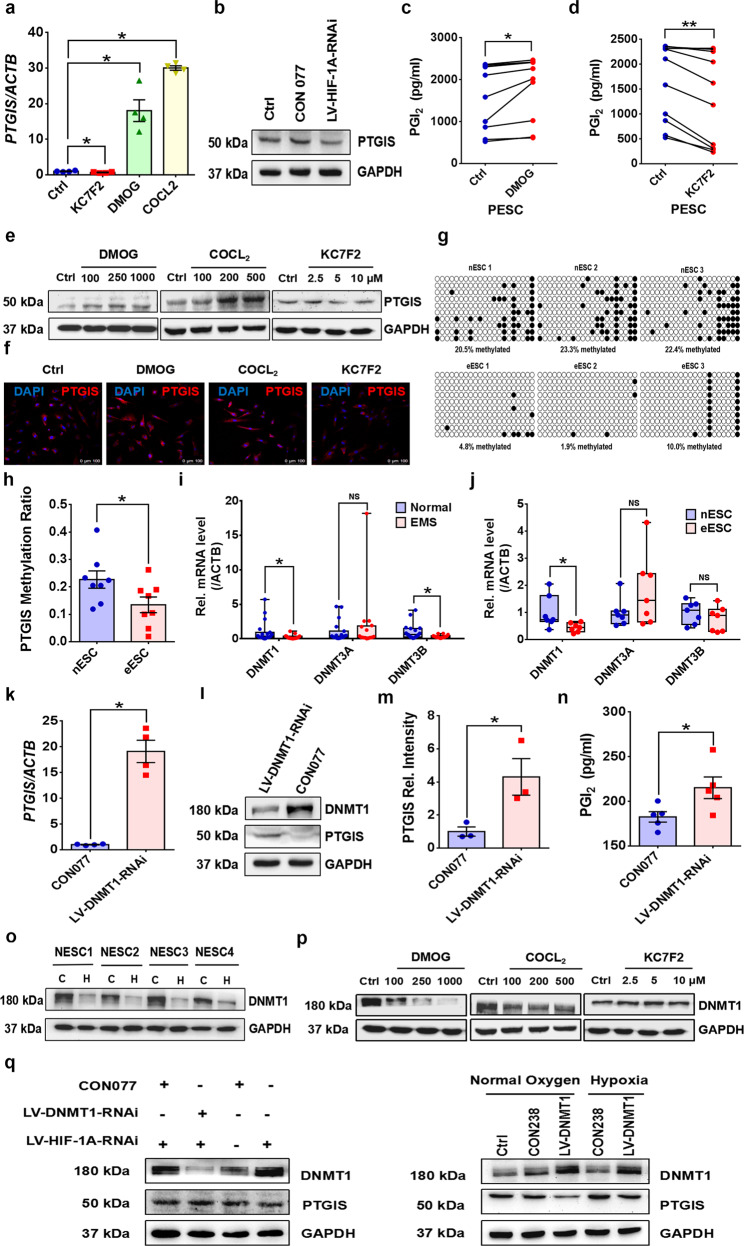
Hypoxia promotes PTGIS/PGI_2_ expression through a DNMT1-mediated methylation deficiency in the PTGIS promoter. **a** When compared to control (Ctrl) treatment, DMOG (250 μM, 48 h) and COCL_2_ (200 μM, 48 h) treatment promoted *PTGIS* expression in HESCs, and KC7F2 (5 μM, 48 h) produced opposing effects. **b** Knocking down HIF-1α depressed the expression of PTGIS in HESCs. **c**, **d** DMOG (250 μM, 48 h) promoted and KC7F2 (5 μM, 48 h) hindered the production of PGI_2_ in PESCs (*n* = 10). **e** DMOG (100, 250 and 1000 μM) (48 h) and COCL_2_ (100, 200 and 500 μM) (48 h) treatment promoted PTGIS expression in a concentration-dependent manner, and KC7F2 (2.5, 5, and 10 μM) (48 h) treatment produced opposing effects. **f** DMOG (250 μM, 48 h) and COCL_2_ (200 μM, 48 h) treatment promoted PTGIS expression, and KC7F2 (5 μM, 48 h) produced opposing effects. **g**, **h** The methylation of the *PTGIS* promoter was decreased in eESCs (*n* = 8) compared to nESCs (*n* = 8). **i** The expression levels of *DNMT1* and *DNMT3B* were reduced in endometriotic lesions, while the level of *DNMT3A* was not significant different. **j** The mRNA level of DNMT1 was decreased in eESCs, while the levels of DNMT3A and DNMT3B did not show significant differences. **k**–**n** Knocking down DNMT1 expression in HESCs promoted PTGIS expression and PGI_2_ production. **o** When compared to Ctrl treatment (normoxic condition), 2% oxygen (hypoxic condition) treatment obviously hindered *DNMT1* expression in PESCs. **p** DMOG (100, 250 and 1000 μM) (48 h) and COCL_2_ (100, 200 and 500 μM) (48 h) treatment hindered DNMT1 expression in HESCs, and KC7F2 (2.5, 5 and 10 μM) (48 h) produced opposing effects in a concentration-dependent manner. **q** Knocking down the expression level of HIF-1α elevated the level of DNMT1 and hindered the expression of PTGIS, and downregulating DNMT1 partly reversed these effects. Overexpression of DNMT1 in HESCs partly reversed the high expression of PTGIS induced by hypoxia. Statistical analysis was performed with Student’s t test, one-way analysis of variance (ANOVA) or the Mann–Whitney test. NS: no significant difference, *P < 0.05, **P < 0.01. HIF-1α hypoxia-inducible factor 1 alpha, WB western blotting, HESCs human endometrial stromal cells, PESCs primary endometrial stromal cells. Scale bar: 100 μm, white dots: unmethylated regions, black dots: methylated regions. C: control group, refers to the normoxic condition; H: 2% oxygen group, refers to the hypoxic condition.

To uncover the mechanism underlying the elevated PTGIS/PGI_2_ in EMs, the microenvironment of disease-modified lesions was investigated. It is widely accepted that endometriotic lesions are in a state of hypoxia^32–34^, and we determined the hypoxia-inducible factor 1 alpha (HIF-1α) levels in all samples. Consistent with a previous study, HIF-1α was extensively augmented in EMs (Supplementary Fig. 1c–g). HESCs were treated with DMOG and COCL_2_ to simulate the hypoxic milieu in EMs, and the results showed that DMOG and COCL_2_ accelerated PTGIS/PGI_2_ production in a concentration-dependent manner; KC7F2 produced opposing effects (Fig. 2a, c–f). Moreover, downregulating the expression of HIF-1α in HESCs hindered PTGIS expression (Fig. 2b, Supplementary Fig. 1h), implicating hypoxia as a critical mediator in PTGIS/PGI_2_ expression promotion. More importantly, epigenetic modification, such as DNA methylation, has also been emphasized to regulate PTGIS expression^35–37^. Thus, we analyzed the methylation status of the *PTGIS* promoter in 3 existing EMs-related GEO datasets (GSE73950, GSE87621 and GSE47361). The methylation of the *PTGIS* promoter in EMs specimens was extensively inhibited (Supplementary Fig. 2a–c), which was consistent with our verification that ovarian ESCs exhibited a deficient methylation level in the *PTGIS* promoter compared to normal ESCs (Fig. 2g, h). To examine the mechanism underlying this phenomenon, we measured the expression levels of the 3 main DNA methyltransferases, DNMT1, DNMT3A and DNMT3B, in tissues and primary ESCs and found a significant reduction in the DNMT1 level but no significant differences in the DNMT3A and DNMT3B levels in EMs (Fig. 2i, j, Supplementary Fig. 1i), implying that the deficient methylation of the *PTGIS* promoter was favored by hampered DNMT1 expression. These results were further confirmed by knocking down *DNMT1* in HESCs, which led to substantially increased levels of PTGIS/PGI_2_ (Fig. 2k–n, Supplementary Fig. 1j), indicating that the abnormal PTGIS/PGI_2_ levels in EMs were also modulated by the DNMT1-mediated methylation deficiency in the PTGIS promoter. As two regulatory pathways were identified in our study, we hypothesized that there might be some correlation between hypoxia and DNMTI, as it has been reported that hypoxia destabilizes DNMT1 expression in ESCs^38^. Thus, a hypoxic milieu was created in vitro by culturing PESCs in a 2% oxygen incubator or treating HESCs with DMOG and COCL_2_, and the results revealed that 2% oxygen substantially hampered the expression of DNMT1 in PESCs and that DMOG and COCL_2_ treatment inhibited DNMT1 expression in a concentration-dependent manner (Fig. 2o, p). Moreover, KC7F2 treatment promoted DNMT1 expression (Fig. 2p). Mechanistically, knocking down DNMT1 in HESCs rescued PTGIS expression, which was inhibited by HIF-1α deficiency, and overexpressing DNMT1 in HESCs partly reversed the high expression level of PTGIS induced by hypoxia (Fig. 2q), indicating that hypoxia promoted PTGIS/PGI_2_ production through the DNMT1-mediated deficient methylation status of the PTGIS promoter.

### PTGIS/PGI_2_ signaling accelerates EMs progression in vivo

**Fig. 3:**
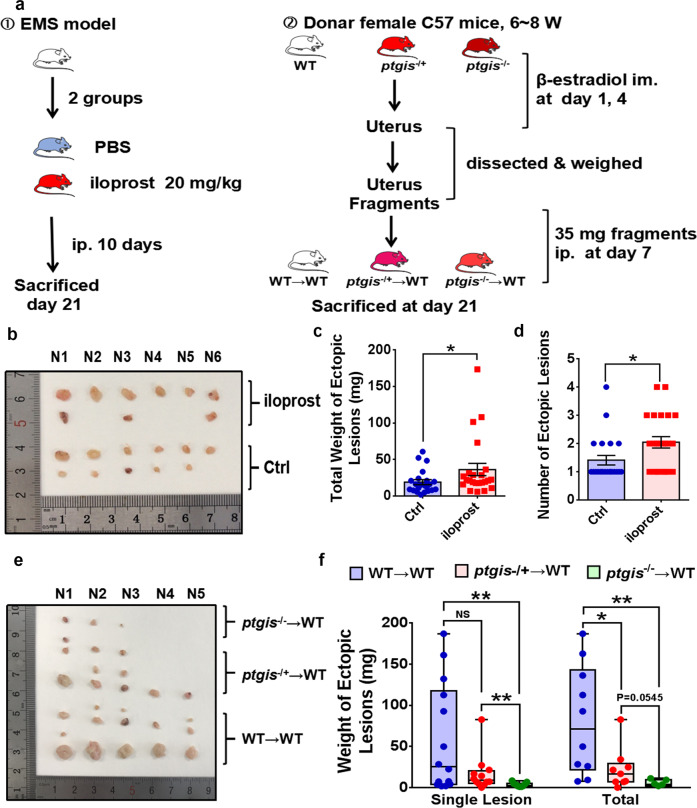
The PTGIS/PGI_2_ signaling pathway accelerates EMs progression in vivo. **a** Schematic of the EMs model establishment process. **b** Collected ectopic lesions in the abdominopelvic cavity of EMs model mice. **c**, **d** Iloprost (a stable PGI_2_ analog) treatment (20 mg/kg) (*n* = 23) increased the weight and number of ectopic lesions in EMs model mice compared to control mice (*n* = 22). **e** Collected ectopic lesions from 3 groups of EMs model mice. **f** The weights of *ptgis*^−/−^ (*n* = 7) and *ptgis*^−/+^ (*n* = 9) uterine debris-originated ectopic lesions were decreased compared to those of lesions in the WT group (*n* = 10). Statistical analysis was performed with Student’s t test, one-way analysis of variance (ANOVA) or the Mann–Whitney test. NS no significant difference, *P < 0.05, **P < 0.01. WT wild type, *ptgis*^−/+^ heterozygote, *ptgis*^−^^/−^ homozygote.

Since increased production of PTGIS/PGI_2_ was present in endometriotic lesions, we investigated the roles of PTGIS/PGI_2_ in EMs. Specifically, we carried out in vivo experiments, and well-established EMs models were separated into iloprost (a stable PGI_2_ analog) and vehicle (PBS) groups. Iloprost at the manufacturer’s recommended doses or PBS was injected intraperitoneally into EMs model mice, and the iloprost group exhibited an increased number and weight of endometriotic lesions (Fig. 3a–d), indicating that the increased PGI_2_ concentration in the mouse peritoneal cavity facilitated EMs progression. Moreover, to precisely decipher the function of the aberrant level of PTGIS in disease-modified endometriotic lesions, we constructed *ptgis*^−/−^ mice, and the PGI_2_ levels in the peritoneal lavage fluid and plasma of male and female *ptgis*^−/−^ mice were considerably reduced (Supplementary Fig. 2d, e). *Ptgis*^−/−^ mice were used as donors for intraperitoneal injection of uterine fragments into recipient mice to establish EMs models. As shown, the EMs model mice that received *ptgis*^−/−^ uterine fragments had dramatically attenuated weights of endometriosis-like lesions in the peritoneal cavity compared to the recipient mice in the WT group (Fig. 3e, f), demonstrating that the activated PTGIS/PGI_2_ signaling pathway facilitated EMs progression in vivo.

### Hypoxia promotes CD16^−^ NK-cell differentiation by activating PTGIS/PGI_2_ signaling

**Fig. 4:**
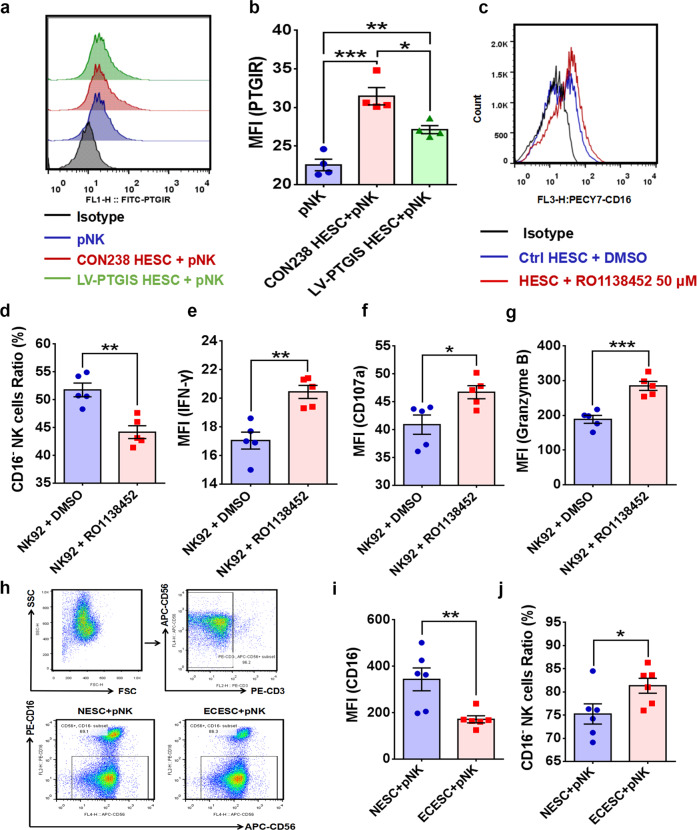
ESCs promote CD16^−^ NK-cell differentiation through PTGIR. **a** PTGIR was expressed on the surface membrane of NK cells. **b** The expression level of PTGIR in NK cells increased after coculture with HESCs. **c**–**g** Treatment with the PTGIR antagonist RO1138452 (50 μM) decreased the percentage of CD16^−^ NK92 cells and promoted CD107a, IFN-ɣ and granzyme B expression. **h** The purity of pNK cells isolated from the peripheral blood of volunteers and the expression level of CD16 were determined by FCM. **i**, **j** The CD16^−^ pNK-cell subpopulation increased and expression level of CD16 reduced after coculture with ectopic ESCs. Statistical analysis was performed with Student’s t test, one-way analysis of variance (ANOVA) or the Mann–Whitney test. *P < 0.05, **P < 0.01, ***P < 0.001. PTGIR PGI_2_ receptor, RO1138452 PTGIR antagonist, MFI mean fluorescence intensity, IFN-ɣ interferon gamma, NESC normal endometrial stromal cells, ECESC ectopic endometrial stromal cells, pNK peripheral NK cells, FCM flow cytometric analysis.

To uncover the mechanism by which the activated PTGIS/PGI_2_ signaling pathway accelerates EMs progression, we considered two critical factors, namely, intrinsic disorders of ectopic ESCs and dysfunctional immunosuppression. RNA-sequencing results showed that PTGIS mainly induced substantially augmented expression of adhesion molecules in HESCs, with other pathways showing no significant differences (Supplementary Fig. 3a–d), and the adhesive ability was considerably enhanced in PTGIS-overexpressing HESCs (Supplementary Fig. 3e, f), suggesting that the PTGIS/PGI_2_ signaling pathway triggered EMs by strengthening the adhesive ability of ESCs. On the other hand, we investigated the roles of PTGIS/PGI_2_ in reprogramming the immune milieu in the peritoneal cavity of EMs patients, and then we analyzed the PTGIS levels in all peritoneal fluid cells collected from healthy and EMs volunteers. Among all peritoneal fluid cells, NK cells had the lowest expression level of PTGIS, while macrophages had the highest, with ESCs having a PTGIS expression level similar to that of as macrophages and much higher than that of NK cells (Supplementary Fig. 4a–e). Thus, we speculated that ectopic ESC-derived PGI_2_ might have considerable influence on the function of NK cells. Moreover, we determined the PGI_2_-specific membrane superficial receptor (PI, PTGIR) levels in NK cells, and the results showed that PTGIR was actually expressed on the surface membrane of NK cells (Fig. 4a, b). Additionally, treatment with the PTGIR antagonist RO1138452 favored enlargement of the CD16^+^ NK92 cell subpopulation and promoted CD107a, interferon gamma (IFN-ɣ) and granzyme B expression (Fig. 4c–g), indicating that blocking PTGIR was of great importance in activating NK cells and that ESC-derived PGI_2_ indeed repressed NK-cell activity.

**Fig. 5:**
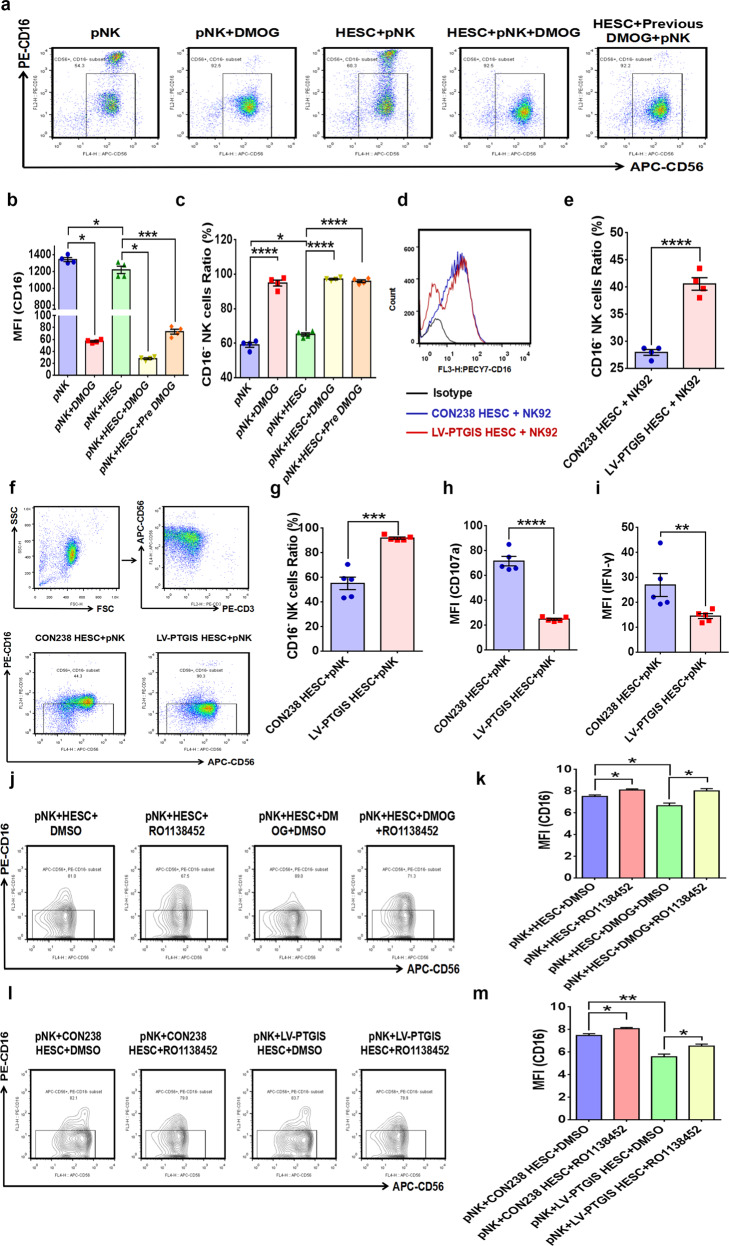
Hypoxia-conditioned ESCs promote CD16^−^ NK-cell differentiation by inducing PGI_2_ production, which can be partly reversed by RO1138452. **a**–**c** Hypoxia-conditioned ESCs accelerated CD16^−^ NK-cell differentiation and hindered the expression of CD16 in NK cells. **d**, **e** Upregulating the expression of PTGIS in HESCs promoted CD16^−^ NK92 cell differentiation. **f**–**i** Upregulating the expression of PTGIS in HESCs accelerated CD16^−^ pNK-cell differentiation and inhibited CD107a and IFN-ɣ expression. **j**, **k** RO1138452 (50 μM) treatment rescued NK cells from the inhibitory effect of DMOG (250 µM) on CD16 expression. **l**, **m** Upregulation of PTGIS in HESCs inhibited the expression of CD16 in pNK cells, and RO1138452 (50 μM) partly rescued this effect. Statistical analysis was performed with Student’s t test, one-way analysis of variance (ANOVA) or the Mann–Whitney test. *P < 0.05, **P < 0.01, ***P < 0.001, ****P < 0.0001.

**Fig. 6:**
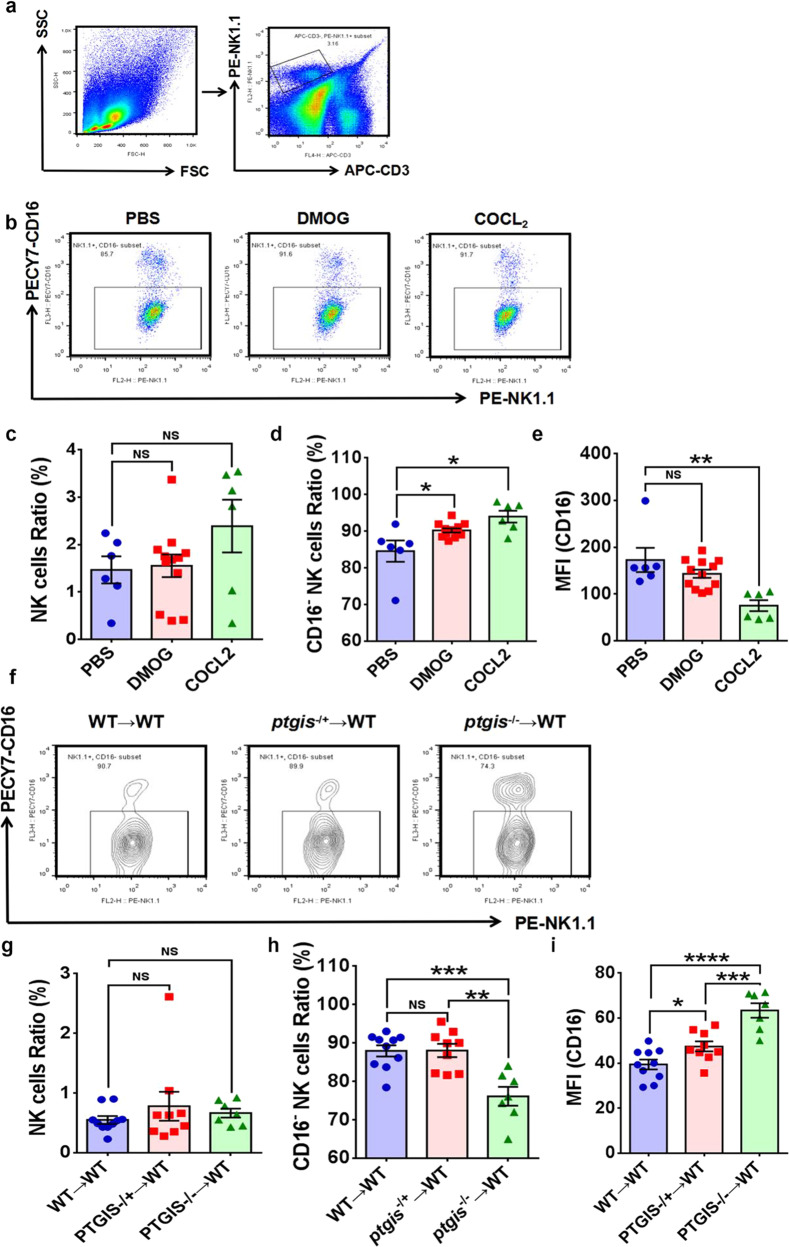
PTGIS/PGI_2_ originating from ESCs promotes CD16^−^ NK-cell differentiation in vivo. **a** NK cells in the peritoneal lavage fluid of EMs model mice were analyzed by FCM. **b**–**e** DMOG (20 mg/kg and 40 mg/kg) and COCL_2_ (40 mg/kg) treatment promoted CD16^−^ NK-cell differentiation in the peritoneal cavity of EMs model mice but had no influence on the NK-cell ratio. **f**–**i** *Ptgis*^−/−^ ectopic lesions in EMs model mice hindered CD16^−^ NK-cell differentiation and increased the CD16 expression level of NK cells in the peritoneal fluid of EMs model mice, but the NK-cell ratio was not significantly different among the three groups. Statistical analysis was performed with Student’s t test, one-way analysis of variance (ANOVA) or the Mann–Whitney test. NS no significant difference, *P < 0.05, **P < 0.01, ***P < 0.001, ****P < 0.0001. WT wild type, *ptgis*^−/+^ heterozygote, *ptgis*^−/−^ homozygote.

In a previous study, we reported that ESCs promoted CD16^−^ pNK-cell differentiation when ESCs were cocultured with pNK cells, and ovarian ESCs exerted more obvious effects^31^, which was further validated in this study (Fig. 4h–j). Based on the results showing that the PTGIR antagonist RO1138452 favored the differentiation of CD16^+^ NK cells, we speculated that the enlarged subpopulation of CD16^−^ NK cells observed after coculturing ESCs with NK cells was ascribed to the function of ESC-derived PGI_2_. Consistent with our speculation, the level of PTGIR increased when NK cells were cocultured with HESCs (Fig. 4b), and we found that DMOG-conditioned HESCs promoted the differentiation of CD16^−^ NK cells when cocultured with pNK cells (Fig. 5a–c). PTGIS-overexpressing HESCs also had similar effects when they were cocultured with NK92 or pNK cells (Fig. 5d, e). Furthermore, the expression of NK-cell activators, CD107a and IFN-ɣ was decreased when PTGIS-overexpressing HESCs were cocultured with pNK cells (Fig. 5f–i), and RO1138452 reversed the promotive effects of HESCs on CD16^−^ NK-cell differentiation (Fig. 5j–m), demonstrating that hypoxia-modified HESCs promoted CD16^−^ NK-cell differentiation through induced PTGIS/PGI_2_ production via activating PTGIR. More importantly, the role of PTGIS/PGI_2_ in regulating NK-cell differentiation was also observed in vivo. Specifically, DMOG and COCL_2_ treatment accelerated EMs progression and promoted CD3^−^NK1.1^+^CD16^−^ (CD16^−^) NK-cell differentiation in mouse models (Supplementary Fig. 5a–d, Fig. 6a–e). In addition, *ptgis*^−/−^ uterine fragments that survived in the abdominal cavity of recipient mice attenuated the differentiation of CD16^−^ NK cells, with no significant difference in NK-cell percentages (Fig. 6f–i), which was consistent with the results of the in vitro experiments.

### CD16^−^ NK cells accelerate EMs progression in vivo

**Fig. 7:**
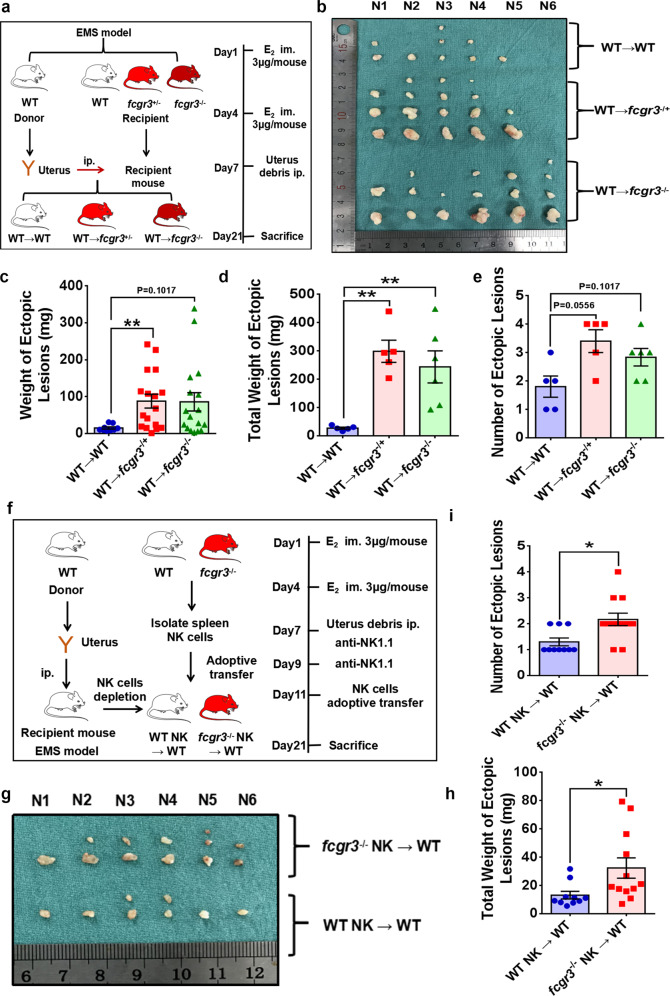
Enlargement of the CD16^−^ subpopulation of NK cells accelerates EMs progression in vivo. **a** EMs model instruction process. **b** Ectopic lesions collected from groups of EMs model mice. **c**, **d** The weight of every ectopic lesion and total weight of ectopic lesions in *fcgr3*^−/+^ and *fcgr3*^−/−^ EMs model mice were increased. **e** The number of ectopic lesions was increased in *fcgr3*^−/+^ and *fcgr3*^−/−^ EMs model mice, but the difference was not significant. **f** Process of adoptively transferring spleen NK cells. **g** Ectopic lesions collected from the WT and *fcgr3*^−/−^ groups of EMs model mice. **h** The total weight of ectopic lesions was elevated in the *fcgr3*^−/−^ group (*n* = 12) compared to the WT group (*n* = 10). **i** *Fcgr3*^−/−^ EMs model mice had an elevated number of ectopic lesions. Statistical analysis was performed with Student’s t test, one-way analysis of variance (ANOVA) or the Mann–Whitney test. *P < 0.05, **P < 0.01. WT wild type, *fcgr3*^−/+^ heterozygote, *fcgr3*^−/−^ homozygote.

**Fig. 8:**
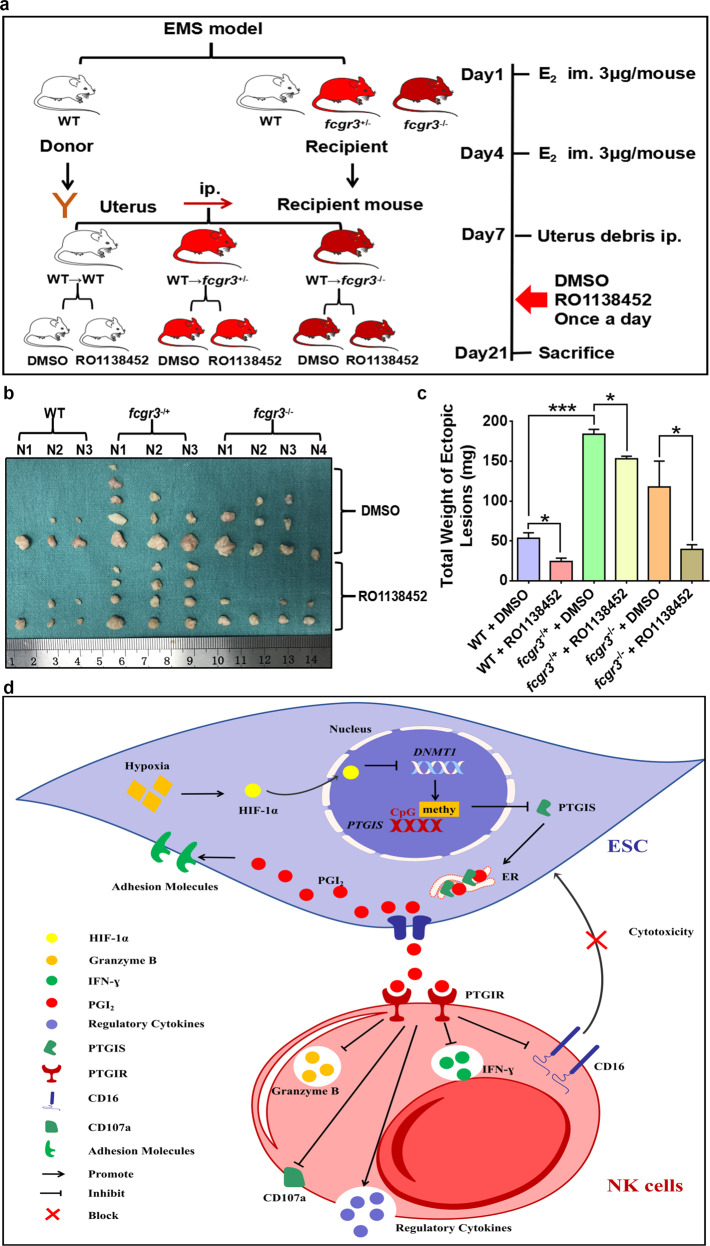
RO1138452 partly attenuates EMs progression promoted by CD16 deficiency in NK cells in vivo. **a** Process of EMs model establishment and treatment. **b** Ectopic lesions collected from groups of EMs model mice. **c** RO1138452 (800 mg/kg) treatment attenuated the increase in the weight of ectopic lesions resulting from CD16 deficiency in NK cells in vivo. **d** Schematic presenting the molecular mechanism by which PTGIS/PGI_2_ promotes EMs progression. Statistical analysis was performed with Student’s t test, one-way analysis of variance (ANOVA) or the Mann–Whitney test. *P < 0.05, ***P < 0.001. WT wild type, *fcgr3*^−/+^ heterozygote, *fcgr3*^−/−^ homozygote.

Our study confirmed that ESCs induced CD16^−^ NK-cell differentiation, but the function of this subpopulation of NK cells in vivo, especially in EMs, remains uncertain. Then, we constructed *fcgr3*^−/−^ mice, and both WT and *fcgr3*^−/−^ mice were used as recipients and treated with RO1138452 or PBS plus DMSO for 10 days. The number and weight of endometriotic lesions in the abdominal cavity of *fcgr3*^−/−^ recipient mice were extensively increased, and RO1138452 partially rescued these effects (Figs. 7a–e, 8a–c), which indicated an immunosuppressive milieu in the abdominal cavity of *fcgr3*^−/−^ mice. This phenomenon might partly be ascribed to decreased NK-cell cytotoxicity. To exclude any influence of other types of immune cells, we depleted NK cells in the abdominal cavity of EMs model mice by intraperitoneally injecting anti-NK1.1, and the depletion efficiency was verified (Supplementary Fig. 6b, c). We isolated spleen NK cells from WT and *fcgr3*^−/−^ mice and divided the well-established peritoneal NK-cell-depleted EMs model mice into two groups. Then, we adoptively transferred isolated spleen NK cells isolated from WT or *fcgr3*^−/−^ mice into the abdominal cavity of the two groups of EMs model mice. Ten days later, the number and weight of endometriotic lesions and the percentage of CD16^−^ NK cells were increased in the EMs model mice adoptively transferred with *fcgr3*^−/−-^ spleen NK cells (Fig. 7f–i, Supplementary Fig. 6a, d, e), suggesting that the CD16^−^ NK-cell subpopulation cells substantially favored the deterioration of EMs.

## Discussion

Molecules involved in arachidonic acid metabolism, such as COX-2 and PGE_2_, have been clearly identified to be critical modulators in EMs, emphasizing that lipid mediators may potentially play some roles in this disease. Herein, we identified another arachidonic acid metabolite, PGI_2_, which was extensively augmented in EMs. Notably, our findings revealed the contribution of elevated PTGIS expression to PGI_2_ overproduction in endometriotic lesions, and the regulatory mechanism underlying the increased PTGIS levels attracted our attention. Although PTGIS has been reported to be predominantly located in ovarian and bladder tissues, GEO datasets provided an informative setting for analyzing PTGIS expression levels in all types of EMs, and we excluded the possible bias resulting from the tissue of origin. As several studies have reported, the expression of COX-2 was increased in endometriotic-like loci^39,40^, and as a downstream enzyme of COX-2, aberrant PTGIS expression might be promoted by COX-2. Nevertheless, research on PTGIS is limited at present, and gene variants^41^, targeted repression of miR-199a/b^42^ and miR-140-3p.1^43^ and genetic modifications, such as DNA methylation, have been reported to contribute to PTGIS expression^35–37^. We identified a deficient methylation status for the PTGIS promoter in EMs, which was mediated by hypoxia/DNMT1; however, the mechanism by which hypoxia modulates DNMT1 expression remains unclear. Several papers have reported that hypoxia destabilizes DNMT1 transcription through HIF1α/2α^44,45^, the AU-rich element binding factor 1 (AUF1)/miR-148a axis^38^, the PI3K/Akt-DNMT1-p53 pathway^46^, and PAS domain-containing protein 1 (EPAS1) protein transactivation^47^, which could provide some clues. Additionally, other regulatory mechanisms independent of DNA methylation may also contribute to hypoxia-induced PTGIS expression. Notably, it has been reported that HIF-1α directly binds to the HRE sequence in the PTGIS promoter region^37^, but whether this mechanism is relevant in EMs requires further exploration.

Using RNA-sequencing analysis, PTGIS-overexpressing HESCs were shown to exhibit an enhanced adhesive ability, indicating that the elevated PTGIS level in ESCs contributed to the early implantation of endometrial debris. Furthermore, this reprogrammed cellular phenotype resulting from increased PTGIS levels or PGI_2_ overproduction is controversial and equivocal. Research on PGI_2_ has substantially concentrated on immunity regulation and vasodilation modulation, while the role of PGI_2_ in remodeling the cell phenotype has been relatively less studied. It has been reported that iloprost promotes myotube formation, migration, and fusion^48^ and that PTGIR and PPARs trigger white to brown adipocyte conversion^49^. However, an emerging role for PTGIS in modulating cell phenotypes has been proposed in recent years; specifically, it was reported that PTGIS hinders hepatic stellate cell activation, induces apoptosis-related protein expression and alleviates CCL4-mediated liver fibrosis^35^. Additionally, PTGIS was associated with liver hepatocellular carcinoma and with liver metastasis in colorectal cancer^50–52^. Knocking down the expression level of PTGIS was shown to promote the migration and proliferation of bladder cancer cells^37^, demonstrating a more complicated role for PTGIS. In addition, *ptgis*^−/−^ mice displayed a phenotype of abnormalities in both the cardiovascular and renal systems (http://www.informatics.jax.org/marker/MGI:1097156), and the former was ascribed to a disorder in the vasodilatory function of PGI_2_, while the etiology of the latter remains a mystery, which suggests that PTGIS could perform additional unknown roles beyond our understanding at present. Regarding the mechanism underlying the enhanced adhesive ability of ESCs, it has been proposed that inhibition of PTGIS reduces stat3 activation in human breast cancer cells^53^, that PTGIS regulates the JAK/STAT signaling pathway in macrophages^43^ and that PTGIR regulates the PKA/CREB and PI3K-γ/PKC-ζ/TRB3/AKT pathways in diabetes^54^. Thus, the biological functions of PTGIS/PGI_2_ need to be further investigated, which might provide insight into the mechanism underlying the elevated adhesive molecule expression.

The crosstalk between ESCs and immune cells has been explored, but the linkage remains largely obscure. Our study identified that ESC-derived PGI_2_ contributed to CD16^−^ NK-cell differentiation in a PTGIR-dependent manner and that the CD16^−^ NK-cell subpopulation promoted EMs progression in vivo, which reinforced the widely accepted hypothesis that CD16^−^ NK cells possess a strengthened regulatory function and weakened killing activity; thus, the approach of hindering CD16^−^ NK-cell accumulation in the peritoneal cavity of EMs patients might be considered an immune-based therapy for combating EMs. Furthermore, our study revealed that the HESC–NK-cell direct coculture system promoted PTGIR expression in the NK cells, that PTGIS-overexpressing HESCs unexpectedly exerted a weaker effect than control HESCs and that upregulating the expression level of PTGIS hindered PTGIR expression in HESCs. We speculated that the overproduction and accumulation of PGI_2_ might negatively impact PTGIR expression. However, how PTGIR modulates CD16 expression is still unclear, and the existing informative research is profoundly limited. CD16 is an activating receptor of NK cells; thus, PTGIR-modified NK-cell inactivation could possibly contribute to the downregulation of CD16 expression. As reported by Bryan Simons, elevated CX3CL1 levels in the airways of *ptgir*^−/−^ mice led to the overproduction of IFN-γ in lung NK cells^19^, which implied a potential role for CX3CL1 in regulating PTGIR-mediated CD16^−^ NK-cell differentiation. Uncovering the detailed mechanism might provide a solid foundation for the application of the PTGIR antagonist RO1138452 in treating EMs in the clinic. Additionally, there are several limitations to our work. First, PTGIS expression was lowest in NK cells among all cells in the peritoneal fluid, and whether these results were equivalent to NK cells producing the lowest concentration of PGI_2_ warrants further investigation in the future. Furthermore, whether ESC-derived PGI_2_ influences the proliferation and apoptosis of NK cells remains unknown. In addition, the PTGIS/PGI_2_ signaling pathway may widely affect angiogenic and inflammatory processes, which are also essential factors in EMs development, and substantial work needs to be done to decipher more roles in the future. Last, the hypoxic milieu of endometriotic lesions also exerted a direct effect on NK cells, which should be taken into account alongside the effects of PGI_2_ derived from ESCs.

Overall, as shown in Fig. 8d, the hypoxic milieu of endometriotic foci destabilized the expression of DNMT1 and reduced the methylation of the PTGIS promoter, further promoting the transcription of PTGIS and catalyzing the biosynthesis of PGI_2_. On the one hand, increased levels of PTGIS/PGI_2_ demonstrated a hitherto unknown role in enhancing the adhesive ability of ESCs, which was critical for the survival of endometriotic debris in the early phase. On the other hand, the overproduction of secreted PGI_2_ promoted CD16^−^ NK-cell differentiation through PTGIR, impairing the cytotoxic activity of NK cells, which contributed to immunosuppressive milieu formation in the peritoneal cavity of EMs patients. Together, these two critical factors accelerated EMs progression. In conclusion, we hope that our findings will provide innovative therapeutic approaches for EMs treatment and insight that helps uncover the underlying mechanism of EMs occurrence.

## Supplementary information

Supplementary information
