# Supplementary material for: Hypoxia-hindered methylation of PTGIS in endometrial stromal cells accelerates endometriosis progression by inducing CD16− NK-cell differentiation
Source: Exp Mol Med. 2022 Jul 4;54(7):890–905. doi: 10.1038/s12276-022-00793-1 (PMC9356144; doi:10.1038/s12276-022-00793-1)
Supplement: Supplementary file 1 — Supplementary information [file 12276_2022_793_MOESM1_ESM.pdf]

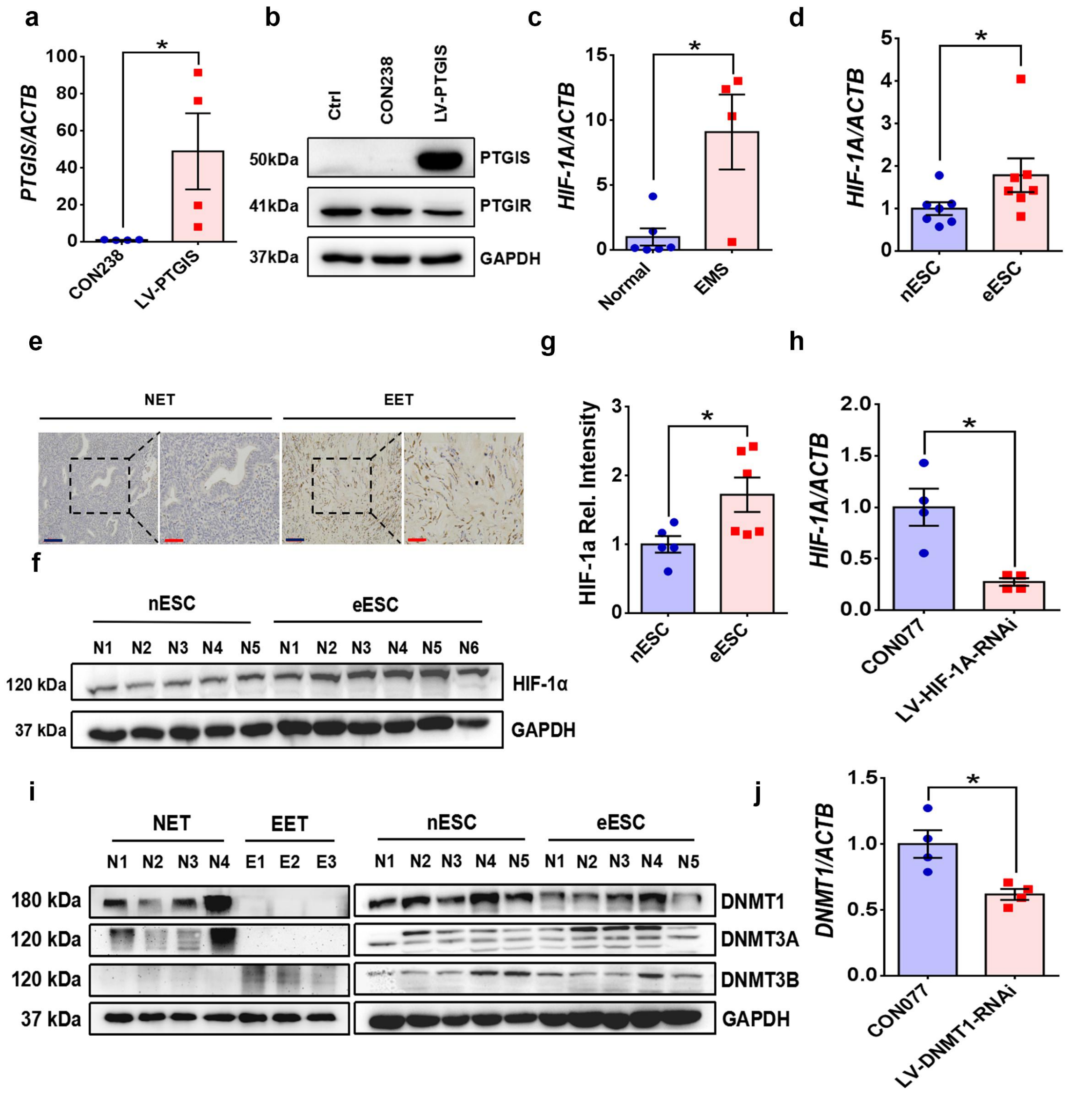

Supplementary Fig. 1: (a-b) Up-regulation expression of PTGIS in HESCs promoted PTGIS expression and hindered PTGIR expression. (c-d) Expression level of *HIF-1A* increased in endometriotic lesions and eESCs. (e) IHC assay displayed enhanced expression of HIF-1α in endometriotic lesions. (f-g) Protein level of HIF-1α augmented in eESCs. (h) Knocking down HIF-1A in HESCs hindered expression level of *HIF-1A*. (i) Protein level of DNMT1 decreased in endometriotic lesions and eESCs. (j) Knocking down DNMT1 in HESCs hampered expression level of *DNMT1*. Statistical analysis was determined by Student's t-test, one-way analysis of variance (ANOVA) or Mann–Whitney test. *P<0.05. Scale: 60 μm (Red) and 100 μm (Blue).

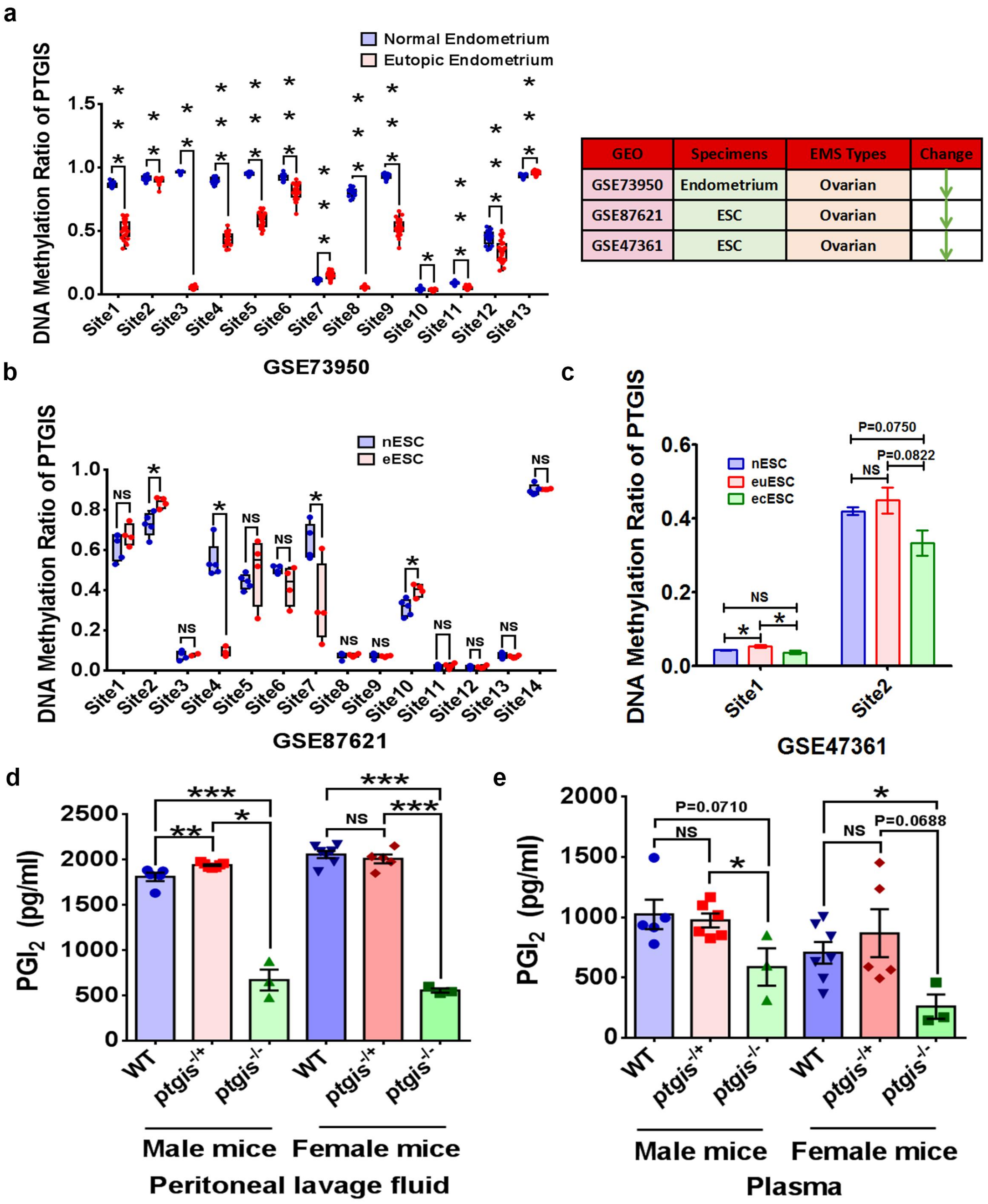

GEO	Specimens	EMS Types	Change
GSE73950	Endometrium	Ovarian	↓
GSE87621	ESC	Ovarian	↓
GSE47361	ESC	Ovarian	↓

Supplementary Fig. 2: Methylation status of PTGIS promoter in GEO database and PGI_2 concentration in peritoneal lavage fluid and plasma of male and female mice. (a) Methylation status of PTGIS promoter decreased in EMs eutopic endometrium in GSE73950. (b) EESCs had inhibited methylation status in PTGIS promoter, when compared to nESCs in GSE87621. (c) Methylation status of PTGIS promoter decreased in eESCs, when compared to nESCs in GSE47361. (d) PGI_2 production condition in peritoneal lavage fluid of male and female mice, and concentration of PGI_2 substantially reduced in *ptgis*$^{-/-}$ mice. (e) Concentration of PGI_2 in plasma of male and female mice, and *ptgis*$^{-/-}$ mice had decreased level of PGI_2, which was determined by ELISA. Statistical analysis was determined by Student's t-test, one-way analysis of variance (ANOVA) or Mann–Whitney test. NS: no significant difference, *P<0.05, **P<0.01, ***P<0.001. Peritoneal lavage fluid: 1 ml of PBS was intraperitoneally injected into peritoneal cavity of each mouse and then the lavage fluid was collected. WT: wild type, *ptgis*$^{-/+}$: heterozygote, *ptgis*$^{-/-}$: homozygote.

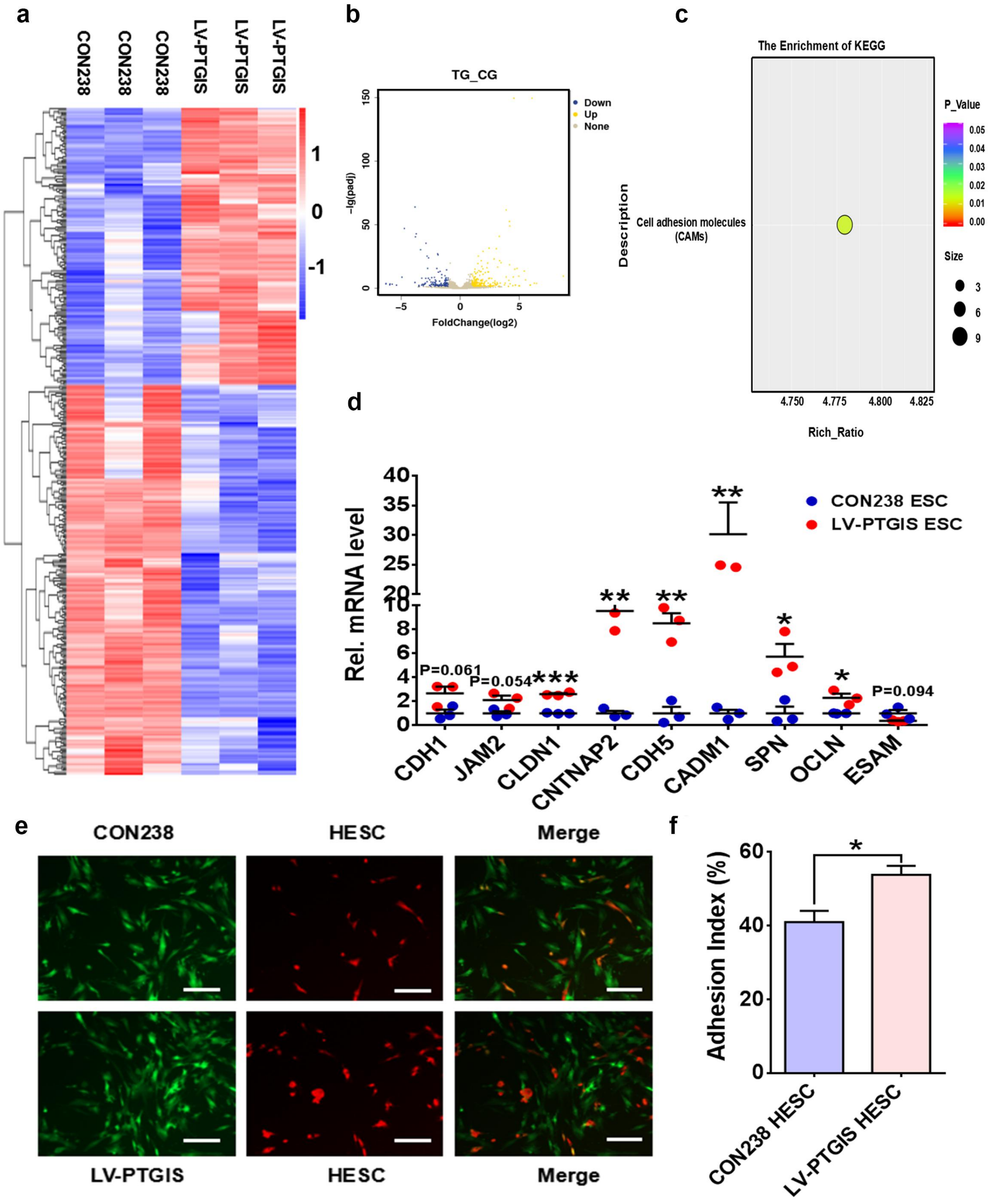

Supplementary Fig. 3: Over-expression of PTGIS elevates adhesion molecules expression and enhances adhesive ability of HESCs. (a-d) Expression level of adhesion molecules increased in PTGIS up-regulated HESCs. (e) Adhesion assay was performed in CON238 and LV-PTGIS HESCs. (f) Adhesion ability increased in PTGIS up-regulated HESCs. Statistical analysis was determined by Student's t-test, one-way analysis of variance (ANOVA) or Mann–Whitney test. NS: no significant difference, *P<0.05, **P<0.01, ***P<0.001. Red fluorescence of HESCs was stained by PKH26, green fluorescence of HESCs came from cells themselves.

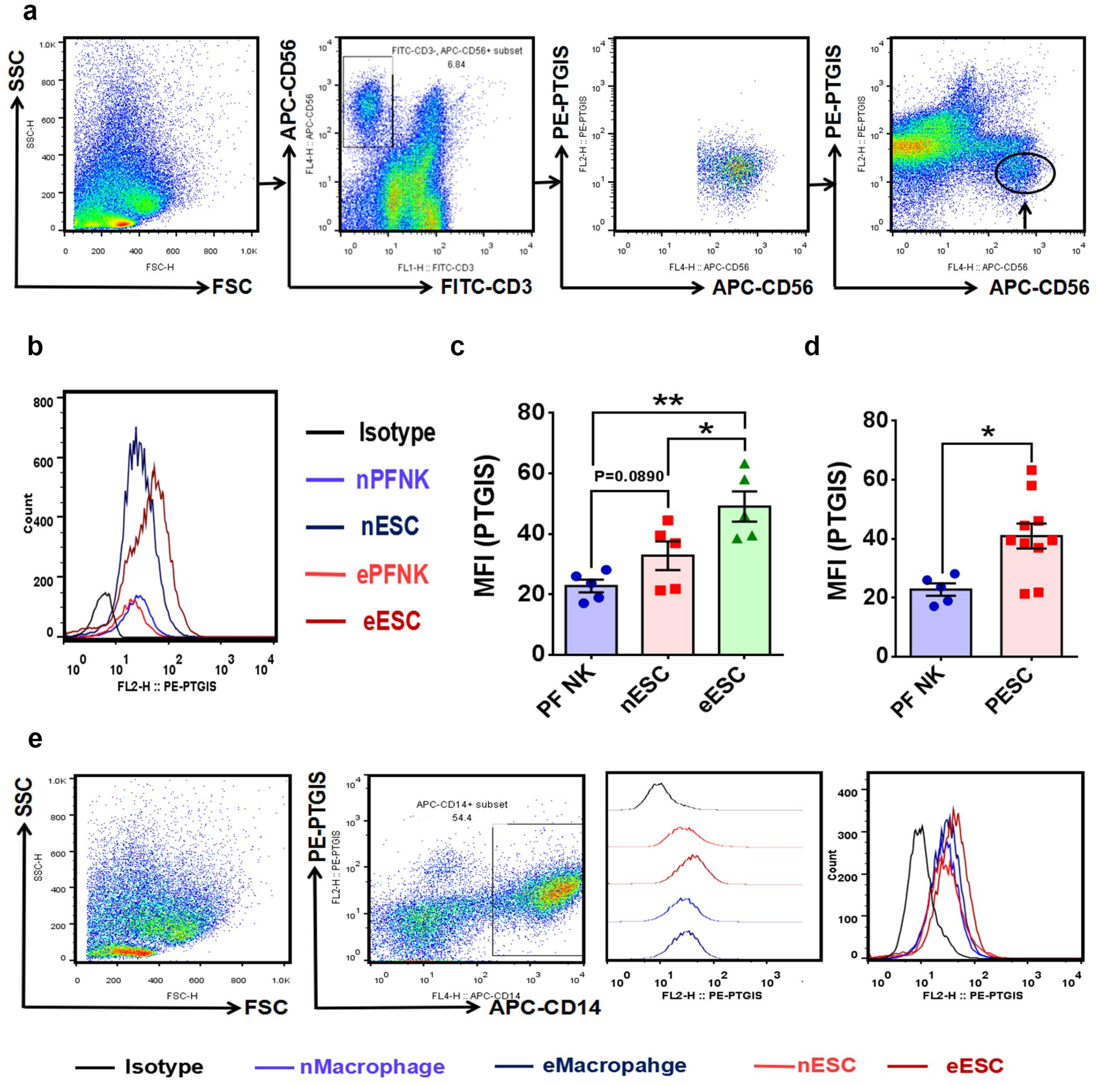

Supplementary Fig. 4: PTGIS expression level in PESCs is higher than NK cells in peritoneal fluid. (a) Expression level of PTGIS in peritoneal fluid NK cells. (b-d) PESCs had higher expression of PTGIS than NK cells. (e) Expression level of PTGIS in macrophage in peritoneal fluid. Statistical analysis was determined by Student's t-test, one-way analysis of variance (ANOVA) or Mann–Whitney test. *P<0.05, **P<0.01. nPFNK: normal peritoneal fluid NK cells, ePFNK: endometriosis peritoneal fluid NK cells, nESC: normal endometrial stromal cells, eESC: ectopic endometrial stromal cells, PESC: primary endometrial stromal cells.

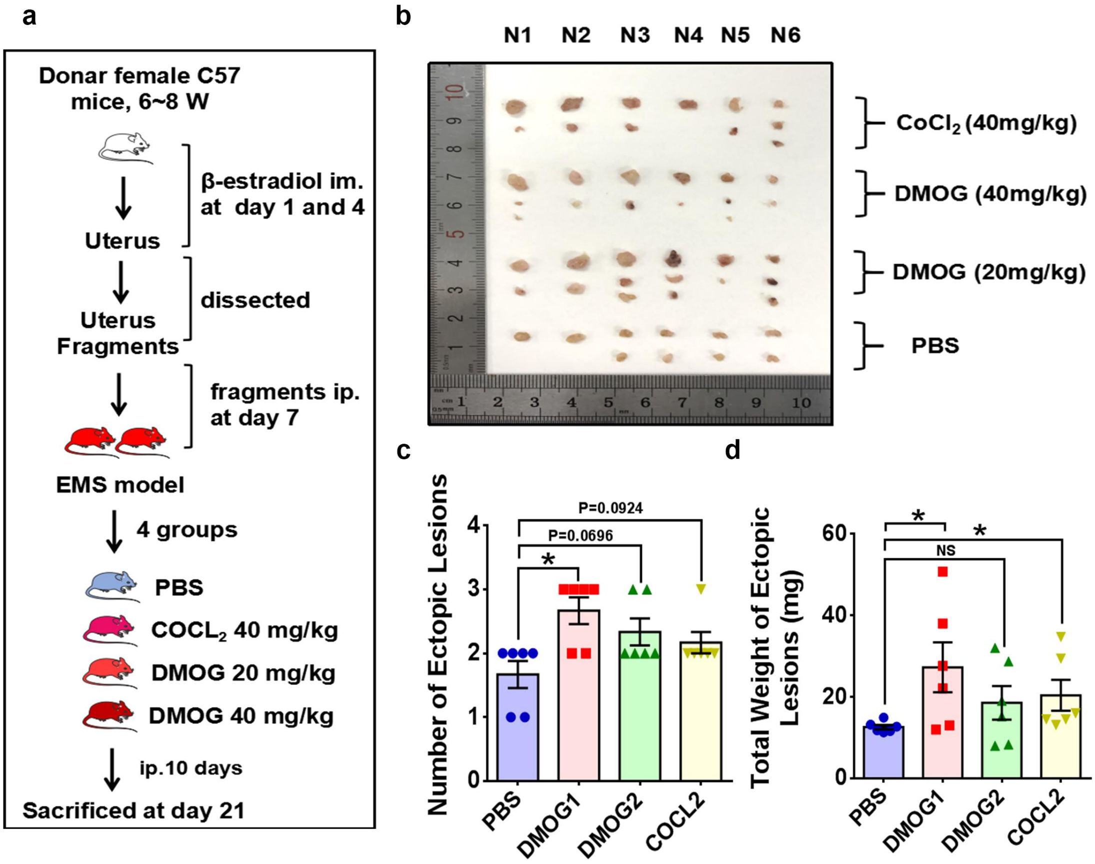

Supplementary Fig. 5: Hypoxia microenvironment accelerates EMs progression in vivo. (a) Ideograph for showing how EMs models were constructed and treatment strategies. (b) Ectopic lesions collected from 4 groups of EMs models. (c-d) DMOG (20 mg/kg) and $COCL_2$ (40 mg/kg) treatment enlarged number and weight of ectopic lesions, whereas 40 mg/kg DMOG treatment had attntuated effects. Statistical analysis was determined by Student's t-test, one-way analysis of variance (ANOVA) or Mann–Whitney test. *P<0.05, NS: no significant difference.

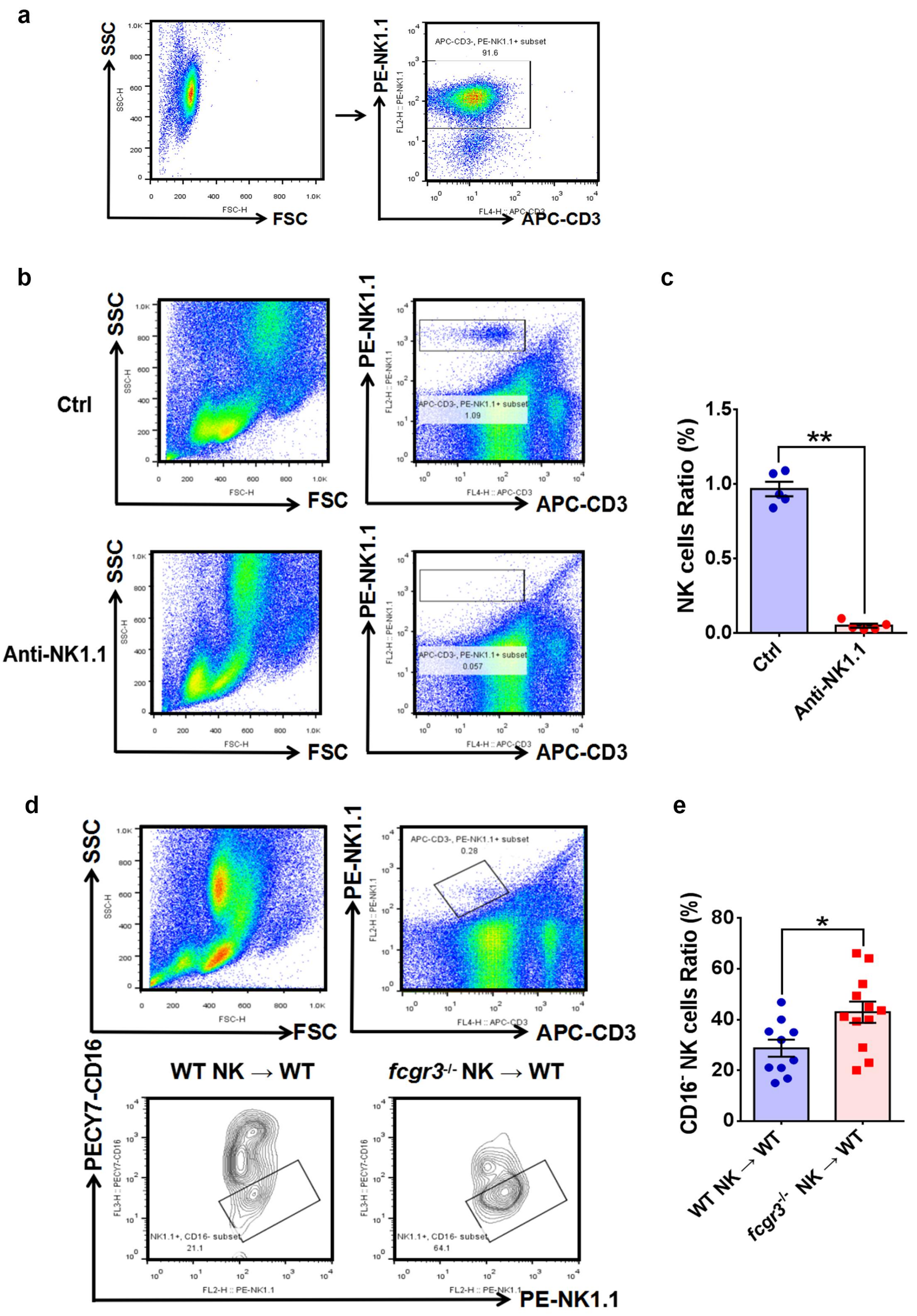

a
SSC
FSC
PE-NK1.1
APC-CD3
APC-CD3-, PE-NK1.1+ subset
91.6
b
c
Ctrl
Anti-NK1.1
APC-CD3-, PE-NK1.1+ subset
1.09
0.057
NK cells Ratio (%)
**
d
e
APC-CD3-, PE-NK1.1+ subset
0.28
WT NK → WT
fcgr3-/- NK → WT
PECY7-CD16
NK1.1+, CD16- subset
21.1
64.1
CD16- NK cells Ratio (%)
*

Supplementary Fig. 6: Anti-NK1.1 treatment significantly deplets NK cells in peritoneal cavity of EMs models. (a) Purity of mouse NK cells isolated from spleen was verified by FCM (percentage of $CD3^-$ $NK1.1^+$ NK cells was larger than 90%). (b) Verificaton of NK cells depletion efficiency (at day 11, 2 days after anti-NK1.1 treatment and before adoptive transferring spleen NK cells). (c) When compared to ctrl group (negative control reagent-injected EMs models), anti-NK1.1 treatment group of EMs models had obviously reduced percentage of NK cells in peritoeal cavity. (d-e) Percentage of $CD16^-$ NK cells increased in *fcgr3*$^{-/-}$ spleen NK cells adoptive transferred EMs models (n=12), when compared to WT group (n=10) (at day 21, 10 days after adoptive transferring spleen NK cells of WT and *fcgr3*$^{-/-}$ mouse). Statistical analysis was determined by Student's t-test, one-way analysis of variance (ANOVA) or Mann–Whitney test. *P<0.05, **P<0.01.

Supplementary Table 1: the clinical information of patients in the study.

Patient Number	Specimens Collected	Diagnosis	Age	Cycle Phase	Pain	Stage (rASRM)
E1	EcEM, eESC	Ovarian EMs	31	Proliferative	Moderate	III
E2	EcEM, eESC	Ovarian EMs	25	Secretory	no	IV
E3	EcEM, eESC	Ovarian EMs	37	Proliferative	no	II
E4	EcEM, eESC	Ovarian EMs	40	Proliferative	no	II
E5	EcEM, eESC	Ovarian EMs	46	Secretory	no	III
E6	EcEM, eESC	Ovarian EMs	43	Proliferative	no	III
E7	EcEM, eESC	Ovarian EMs	26	Proliferative	Mild	II
E8	EcEM, eESC	Ovarian EMs	39	Secretory	no	II
E9	EcEM, eESC	Ovarian EMs	24	Proliferative	Mild	III
E10	EcEM, eESC	Ovarian EMs	33	Proliferative	Mild	III
E11	EcEM, eESC	Ovarian EMs	39	Proliferative	no	III
E12	EcEM, eESC	Ovarian EMs	46	Proliferative	no	III
E13	EcEM	Ovarian EMs	29	Proliferative	Mild	III
E14	EcEM	Ovarian EMs	40	Proliferative	Mild	III
E15	EcEM, EuEM	Ovarian EMs	31	Proliferative	Moderate	IV
E16	EcEM	Ovarian EMs	37	Proliferative	no	III
E17	EcEM	Ovarian EMs	39	Proliferative	no	III
E18	EcEM, EuEM	Ovarian EMs	32	Proliferative	no	III
E19	EcEM, EuEM	Ovarian EMs	27	Proliferative	no	III
E20	EcEM	Ovarian EMs	29	Secretory	no	III
E21	EcEM, EuEM	Ovarian EMs	43	Proliferative	Moderate	IV
E22	EcEM	Ovarian EMs	31	Proliferative	Moderate	III
E23	EcEM	Ovarian EMs	25	Proliferative	Moderate	II
E24	EcEM, EuEM	Ovarian EMs	30	Proliferative	no	III
E25	PF	Ovarian EMs	29	Proliferative	Mild	III
E26	PF	Ovarian EMs	34	Proliferative	Mild	II

E27	PF	Ovarian EMs	30	Proliferative	Mild	II
E28	PF	Ovarian EMs	29	Secretory	Moderate	III
E29	PF	Ovarian EMs	28	Proliferative	Severe	III
E30	PF	Ovarian EMs	29	Proliferative	Mild	II
E31	PF	Ovarian EMs	47	Secretory	Moderate	IV
E32	PF	Ovarian EMs	41	Proliferative	Moderate	II
E33	PF	Ovarian EMs	44	Secretory	no	III
E34	PF	Ovarian EMs	29	Proliferative	Severe	III
E35	PF	Ovarian EMs	38	Proliferative	Mild	III
E36	PF	Ovarian EMs	37	Proliferative	no	III
E37	PF	Ovarian EMs	41	Proliferative	no	III
E38	PF	Ovarian EMs	36	Secretory	no	II
E39	PF	Ovarian EMs	39	Proliferative	no	IV
E40	PF	Ovarian EMs	26	Secretory	no	III
E41	PF	Ovarian EMs	34	Proliferative	no	III
E42	PF	Ovarian EMs	37	Proliferative	Mild	IV
E43	PF	Ovarian EMs	33	Proliferative	no	III
E44	PF	Ovarian EMs	48	Proliferative	Mild	III
E45	PF	Ovarian EMs	28	Proliferative	no	II
E46	PF	Ovarian EMs	25	Secretory	no	IV
E47	PF	Ovarian EMs	28	Proliferative	no	III
E48	PF	Ovarian EMs	38	Proliferative	no	III
E49	PF	Ovarian EMs	42	Proliferative	no	III
E50	PF	Ovarian EMs	37	Proliferative	no	II
E51	PF	Ovarian EMs	35	Proliferative	Moderate	II
E52	PF	Ovarian EMs	36	Secretory	no	III
E53	PF	Ovarian EMs	31	Secretory	Moderate	III
N1	NEM, nESC	Tubal Infertility	31	Proliferative	no	-
N2	NEM, nESC	Tubal Infertility	26	Proliferative	no	-

N3	NEM, nESC	Tubal Infertility	38	Proliferative	no	-
N4	NEM, nESC	Tubal Infertility	36	Secretory	no	-
N5	NEM, nESC	Tubal Infertility	35	Proliferative	no	-
N6	NEM, nESC	Leiomyoma	50	Secretory	no	-
N7	NEM, nESC	Tubal Infertility	30	Proliferative	Mild	-
N8	NEM, nESC	Tubal Infertility	29	Proliferative	no	-
N9	NEM, nESC	Leiomyoma	50	Proliferative	no	-
N10	NEM, nESC	Tubal Infertility	30	Proliferative	Moderate	-
N11	NEM, nESC	Tubal Infertility	40	Proliferative	no	-
N12	NEM, nESC	Leiomyoma	47	Proliferative	no	-
N13	NEM	Leiomyoma	37	Secretory	no	-
N14	NEM	Leiomyoma	38	Proliferative	Moderate	-
N15	NEM	Leiomyoma	30	Proliferative	Moderate	-
N16	NEM	Leiomyoma	40	Proliferative	no	-
N17	NEM	Leiomyoma	46	Secretory	no	-
N18	NEM	Tubal Infertility	33	Proliferative	no	-
N19	NEM	Leiomyoma	42	Secretory	no	-
N20	NEM	Leiomyoma	40	Proliferative	no	-
N21	PF	Leiomyoma	31	Secretory	Mild	-
N22	PF	Leiomyoma	40	Proliferative	no	-
N23	PF	Tubal Infertility	41	Proliferative	no	-
N24	PF	Leiomyoma	49	Secretory	no	-
N25	PF	Leiomyoma	28	Proliferative	no	-
N26	PF	Leiomyoma	37	Secretory	Mild	-
N27	PF	Tubal Infertility	21	Proliferative	Moderate	-
N28	PF	Tubal Infertility	37	Secretory	no	-
N29	PF	Tubal Infertility	29	Proliferative	Moderate	-
N30	PF	Tubal Infertility	36	Proliferative	no	-
N31	PF	Tubal Infertility	41	Proliferative	no	-

N32	PF	Tubal Infertility	25	Proliferative	no	-
N33	PF	Tubal Infertility	28	Proliferative	no	-
N34	PF	Tubal Infertility	27	Proliferative	no	-
N35	PF	Tubal Infertility	35	Proliferative	no	-
N36	PF, PB	Tubal Infertility	35	Proliferative	no	-
N37	PF, PB	Leiomyoma	32	Secretory	Mild	-
N38	PF, PB	Benign Ovarian Cyst	48	Proliferative	Severe	-
N39	PF, PB	Benign Ovarian Cyst	26	Proliferative	Moderate	-
N40	PF, PB	Benign Ovarian Cyst	30	Proliferative	no	-
N41	PF, PB	Benign Ovarian Cyst	29	Proliferative	no	-
N42	PF, PB	Benign Ovarian Cyst	39	Secretory	no	-
N43	PF, PB	Benign Ovarian Cyst	29	Proliferative	Mild	-
N44	PF, PB	Benign Ovarian Cyst	32	Secretory	Moderate	-
N45	PF, PB	Leiomyoma	41	Proliferative	no	-
N46	PF, PB	Leiomyoma	41	Proliferative	Moderate	-
N47	PF, PB	Benign Ovarian Cyst	32	Proliferative	Severe	-

Abbreviation:

rASRM: Revised American Society for Reproductive Medicine classification; EcEM: ectopic endometrium; EuEM: Eutopic endometrium; NEM: normal endometrium; nESC: normal endometrial stromal cells; eESC: ectopic endometrial stromal cells; PF: peritoneal fluid; PB: peripheral blood; EMs: endometriosis.

Supplementary Table 2: The features of reagents.

Reagent	Application	Manufacturer	Catalogue
Anti-HIF-1 alpha	WB	Abcam	ab51608
Anti-PTGIS/PGIS	WB	Abcam	ab23668
Anti-DNMT1	WB	CST	5032
Anti-DNMT3B	WB	CST	67259
Anti-Prostacyclin receptor	WB	Abcam	ab196653
Anti-DNMT3A	WB	Abcam	ab188470
FITC anti-human CD3	FCM	Biolegend	300406
PE anti-human CD16	FCM	Biolegend	302008
APC anti-human CD56 (NCAM)	FCM	Biolegend	318310
FITC anti-mouse CD16/32	FCM	Biolegend	101305
PE anti-mouse NK1.1	FCM	Biolegend	108708
PE/Cy7 anti-human CD16	FCM	Biolegend	302016
Purified anti-mouse NK1.1	FCM	Biolegend	108759
APC anti-mouse CD3	FCM	Biolegend	100236
FITC anti-mouse CD16/32	FCM	Biolegend	101305
FITC anti-human CD107a (LAMP-1)	FCM	Biolegend	328606
PE/Cy7 anti-mouse CD16/32	FCM	Biolegend	101318
PE anti-human/mouse Granzyme B	FCM	Biolegend	372207
PE/Cy7 anti-human IFN-γ	FCM	Biolegend	502527
PE anti-human CD3	FCM	Biolegend	300308
FITC anti-mouse CD107a (LAMP-1)	FCM	Biolegend	121605
FITC anti-human CD3	FCM	Biolegend	300306
Intracellular Staining Perm Wash Buffer	FCM	Biolegend	421002
Fixation Buffer	FCM	Biolegend	420801
NK Cell Isolation Kit, mouse	Cell Isolation	Miltenyi	130-115-818
EasySep Human NK Cell Iso Kit	Cell Isolation	StemCell	17955
MS Separation columns	Cell Isolation	Miltenyi	130-042-201

AutoMACS Running Buffer	Cell Isolation	Miltenyi	130-091-221
DAB Horseradish Peroxidase Color Development Kit	IHC	Biotech Well	WB0167
RIPA	WB	Biotech Well	WB0101
PVDF membrane	WB	Millipore	IPVH00010
CELLSAVING	Cell Culture	NCM	C40050
Iloprost	Reagent	MedChemExpress	HY-A0096
RO1138452	Reagent	MedChemExpress	HY-108912
KC7F2	Reagent	MedChemExpress	HY-18777
DMOG	Reagent	MedChemExpress	HY-15893
$COCL_2$	Reagent	Sigma	7791-13-1
17-β estradiol	Reagent	Sigma	E2758
PrimeScript RT Master Mix	qRT-PCR	Takara	RR036A
TB Green Premix Ex TaqTMII	qRT-PCR	Takara	RR820A
RNAiso Plus	qRT-PCR	Takara	9109
Serum-Free Medium For Lymphocyte	Cell Culture	DAKEWE	6111021
6-keto Prostaglandin F1a ELISA Kit	ELISA	Cayman	515211
Anti-PTGIS/PGIS (PE)	FCM	Abcam	Ab92750
immobilon western chemilum HRP substrate	WB	Millipore	WBKLS0100
